# Gut Microbiota and Metabolite Remodeling Underlies the Anxiolytic Effect of *Anshen Bunao* Oral Liquid

**DOI:** 10.3390/ph19060831

**Published:** 2026-05-26

**Authors:** Yan Chen, Song Lei, Zhipeng Chen, Wenbo Gao, Gang Liu, Yongkuan Wang, Leqi Wang, Xiuyun Zhang, Xue Xiao, Qinqiang Long

**Affiliations:** 1 Guangdong Metabolic Diseases Research Center of Integrated Chinese and Western Medicine, Guangdong Pharmaceutical University, Guangzhou 510006, Chinaxuexiao@gdpu.edu.cn (X.X.); 2 Jilin Aodong Yanbian Pharmaceutical Co., Ltd., Yanbian 133700, China; 3 Jiyuan Neurohealth Industry Research Institute of Guangdong Pharmaceutical University, Guangzhou 510006, China

**Keywords:** *Anshen Bunao* oral liquid, chronic restraint stress (CRS), anxiety disorders, microbiome, untargeted metabolomics

## Abstract

**Background/Objectives**: *Anshen Bunao* Oral Liquid (ABOL) is a traditional medicinal formula comprising *Cornu Cervi Pantotrichum*, *Radix Polygoni Multiflori Preparata* and other ingredients. It replenishes essence, nourishes qi and blood, and soothes the spirit. It is used in clinical practice to treat neurasthenia and insomnia (emotion-related symptoms), and its key component, glycyrrhizin, exhibits anxiolytic properties. This aligns with the holistic approach of traditional Chinese medicine (TCM) to regulating neuropsychiatric disorders. The aim of this study is to evaluate the anxiolytic efficacy of ABOL in rats with anxiety induced by chronic restraint stress (CRS), and to clarify its mechanism by focusing on modulation of the gut–brain axis (microbiota and metabolism). **Methods**: Sprague-Dawley rats underwent three hours of restraint per day for 28 days to induce anxiety. ABOL was administered intragastrically in three doses. Anxiety-like behaviours were assessed using OFT, EPM and SPT. Serum, tissue and faecal samples were analysed using ELISA, histopathology, immunohistochemistry, non-targeted metabolomics, 16S rRNA sequencing and RT-qPCR. **Results**: CRS induced anxiety-like behaviours, impaired weight gain and perturbed the balance of neurotransmitters (decreasing 5-HT, GABA, NE and DA, while increasing CORT), inducing inflammation/oxidative stress, hippocampal neuronal injury, intestinal barrier dysfunction and gut microbiota/metabolic dysregulation. ABOL effectively reversed these abnormalities by restoring the balance of neurotransmitters and the HPA axis, suppressing inflammation and oxidation, protecting neurons and the intestinal barrier, remodelling the gut microbiota (enriching *Akkermansia* and balancing *Firmicutes*/*Bacteroidota*) and regulating sphingolipid and glycerophospholipid pathways. The interaction between the gut microbiota and metabolites may contribute to this pharmacological effect. **Conclusions**: ABOL exerts anxiolytic effects by modulating the gut–brain axis at multiple targets, involving microbiota remodelling, regulation of lipid metabolism and improvement of pathology. This validates its ethnopharmacological value, linking traditional Chinese medicine to the development of modern anxiolytics.

## 1. Introduction

Anxiety disorder is characterised by excessive and unrealistic worry and anxiety that impair daily functioning. One of the most prevalent mental illnesses globally, it is characterised by persistent excessive worry and fear, with clinical manifestations such as chest tightness, sweating, restlessness, dyspnoea, dizziness and palpitations [1]. Historically, it was long classified as a symptom of neurasthenia [2]. From classical times until the late 19th century, anxiety disorder was not recognised as an independent disease. It was not until 1980 that generalised anxiety disorder (GAD) was formally included as a diagnostic category in the Diagnostic and Statistical Manual of Mental Disorders (DSM) [3]. The disorder encompasses several subtypes, including panic disorder with or without agoraphobia, generalised anxiety disorder, social anxiety disorder, specific phobia, and separation anxiety disorder. Epidemiological data indicate that 33.7% of individuals worldwide will be affected by an anxiety disorder at some point in their lives [4]. Due to its high prevalence, recurrence rate and severe complications, the World Health Organization (WHO) has ranked it as the ninth leading cause of health-related disability [5]. Major depressive disorder is the most common comorbidity of GAD, with 56.3% of GAD patients experiencing severe disability [6]. In terms of social costs, GAD significantly impairs work productivity and is a major cause of occupational dysfunction. Additionally, patients with anxiety disorders frequently visit medical institutions, substantially increasing the total societal healthcare burden [7]. Furthermore, the disorder affects individuals across all age groups, with age-specific subtypes: separation anxiety disorder in childhood, social anxiety disorder in adolescence, and generalized anxiety disorder in middle and old age [8].

The pathogenesis of anxiety disorders is complex and multifactorial, involving interactions between multiple biological and environmental factors. Modern medicine suggests that the disorder’s development is associated with hypothalamic–pituitary–adrenal (HPA) axis dysfunction [9], neuroinflammation [10], oxidative stress [11], neurotransmitter imbalance [12], and gut microbiota dysbiosis [13]. In patients with anxiety disorder, HPA axis dysfunction is primarily characterised by impaired glucocorticoid receptor function, which is often accompanied by an increased intensity and duration of HPA axis activation [9]. Neurotransmitter imbalance involves multiple systems, including the glutamatergic, GABAergic, cholinergic, dopaminergic and serotonergic pathways [14]. The central nervous system (CNS) and the gut communicate with each other through a complex regulatory network involving the nervous, endocrine, and immune systems. This network is collectively defined as the brain–gut axis [15]. This bidirectional regulation is primarily mediated by multiple pathways, with neural regulation playing a fundamental role. This communication is achieved neurally through impulse transmission in the autonomic nervous system, including the vagus nerve, afferent fibres, efferent fibres, and the enteric nervous system (ENS) [16]. There is accumulating evidence that vagal neurons are closely involved in mental health and mood-related disorders [17]; for example, Lactobacillus rhamnosus (JB-1) loses its anxiolytic effect in mice with subdiaphragmatic vagotomy [18], highlighting the critical role of the vagus nerve in mediating emotional regulation via the brain–gut axis. In addition to neural regulation, the gut microbiota serves as a key regulator of the brain–gut axis by modulating neurotransmitter homeostasis. Specifically, gut microbes can encode the genes required for enzymes involved in neurotransmitter synthesis, thereby promoting the production of neurotransmitters or their precursors. Alternatively, they can produce neurotransmitters directly; for instance, Lactobacillus produces gamma-aminobutyric acid (GABA), while Escherichia coli generates norepinephrine (NE), serotonin, and dopamine [19]. These microbe-derived neurotransmitters can cross the intestinal mucosa, enter the systemic circulation and subsequently exert regulatory effects on CNS function. Furthermore, dysbiosis of the gut microbiota can disrupt immune system function, and short-chain fatty acids (SCFAs)—a major class of gut microbial metabolite—play a multifaceted role in linking the gut microbiota to CNS function. SCFAs regulate the integrity of the intestinal barrier and gastrointestinal immunity to protect against neuroinflammation, and they also modulate brain functions such as emotional regulation and cognitive processes [20,21]. As the main innate immune cells in the central nervous system (CNS), microglia play a crucial role in immune defence. Gut microbiota can promote microglial homeostasis through short-chain fatty acid (SCFA)-mediated signalling [22], further establishing the regulatory connection between gut microbiota, immunity, and the CNS via the brain–gut axis. The ratio of Firmicutes to Bacteroidetes (F/B) is one of the most classic macroscopic indicators of structural homeostasis in the gut microbiota. An increasing number of studies have confirmed that an imbalanced F/B ratio is closely associated with various metabolic, digestive, neurological and chronic inflammatory diseases [23,24,25], and this correlation may be attributed to impaired intestinal barrier function induced by alterations in microbial community structure. While the correlation between the dysregulation of the F/B ratio and neurological diseases is only partial and the specific pattern of variation remains unclear, this phenomenon highlights the importance of gut microbiota as a potential therapeutic strategy for psychiatric disorders such as anxiety and depression.

Current clinical treatments for anxiety disorders mainly include cognitive behavioural therapy (CBT) and pharmacotherapy [26]. Most anxiolytic drugs act through monoaminergic (predominantly serotonergic) systems, such as selective serotonin reuptake inhibitors, or through amino acid neurotransmitters, such as GABA or glutamate, or through benzodiazepines [27]. However, due to the recurrent nature of anxiety disorders, patients often require long-term medication. The potential side effects of benzodiazepines, such as hepatotoxicity and withdrawal symptoms, render them unsuitable for long-term use. Therefore, the development of safe, effective and low-side-effect alternative therapies is an urgent unmet need. Traditional Chinese medicine (TCM) and its formulations offer advantages such as multi-component, multi-target action, minimal adverse reactions, and suitability for long-term use. Emerging evidence suggests that TCM has promising therapeutic potential in the treatment of anxiety disorders [28], providing new insights and directions for clinical management.

*Anshen Bunao* Oral Liquid (ABOL) is a TCM compound preparation consisting of *Pleuropterus multiflorus* (Thunb.) Turcz. ex Nakai [Polygonaceae], *Zingiber officinale* Roscoe [Zingiberaceae], *Glycyrrhiza uralensis* Fisch. ex DC. [Fabaceae], *Ziziphus jujuba* Mill. [Rhamnaceae], *Epimedium brevicornu* Maxim. [Berberidaceae], *Cornu Cervi Pantotrichum*, and Vitamin B1. The botanical names have passed MPNS verification and were accessed on 2 February 2026. It possesses the pharmacological effects of replenishing essence and marrow, benefiting qi and nourishing blood, and invigorating the brain to soothe the spirit. It is used clinically to alleviate symptoms such as dizziness, fatigue, amnesia, insomnia and neurasthenia caused by kidney essence deficiency and qi-blood insufficiency. Previous studies have demonstrated that ABOL can regulate serum factors to promote the recovery of bone marrow haematopoietic function in a mouse model of qi-blood deficiency [29]. Furthermore, ABOL can effectively alleviate cognitive dysfunction by inhibiting the aggregation of amyloid-β (Aβ) and phosphorylated tau, thereby reducing neuronal damage and improving Alzheimer’s disease [30]. Additionally, ABOL can exert anti-ageing effects by regulating sphingolipid and arachidonic acid metabolism, thereby suppressing inflammation and oxidative stress [31]. In the treatment of neurasthenia, a combination of ABOL and paroxetine has been shown to be more effective than paroxetine monotherapy. Higher levels of brain-derived neurotrophic factor (BDNF) and cortisol (CORT) have been observed in the combined treatment group [32], indicating that ABOL exerts neurotrophic effects, improves negative emotions and facilitates the recovery of neural function. Furthermore, its core components and excipients exhibit potential anxiolytic properties: the main active ingredient of licorice root, glycyrrhizin, promotes glutamate reuptake in the medial prefrontal cortex (mPFC) and alleviates anxiety-like behaviours in mice [33]. B vitamins have also been recognised as an effective and well-tolerated adjuvant strategy for improving depression and anxiety [34]. Given the close association between anxiety disorders, insomnia and neurasthenia, and the pharmacological characteristics and advantages of the components of ABOL, we hypothesise that ABOL may have a regulatory effect on anxiety disorders. This study aims to investigate the ameliorative effect of ABOL on chronic restraint stress-induced anxiety-like behaviour in a rodent model and to determine the underlying mechanism, providing a theoretical basis for the development of novel therapeutic agents for anxiety disorders.

## 2. Results

### 2.1. ABOL Improves Anxiety-like Behavior in Rats

**Figure 1:**
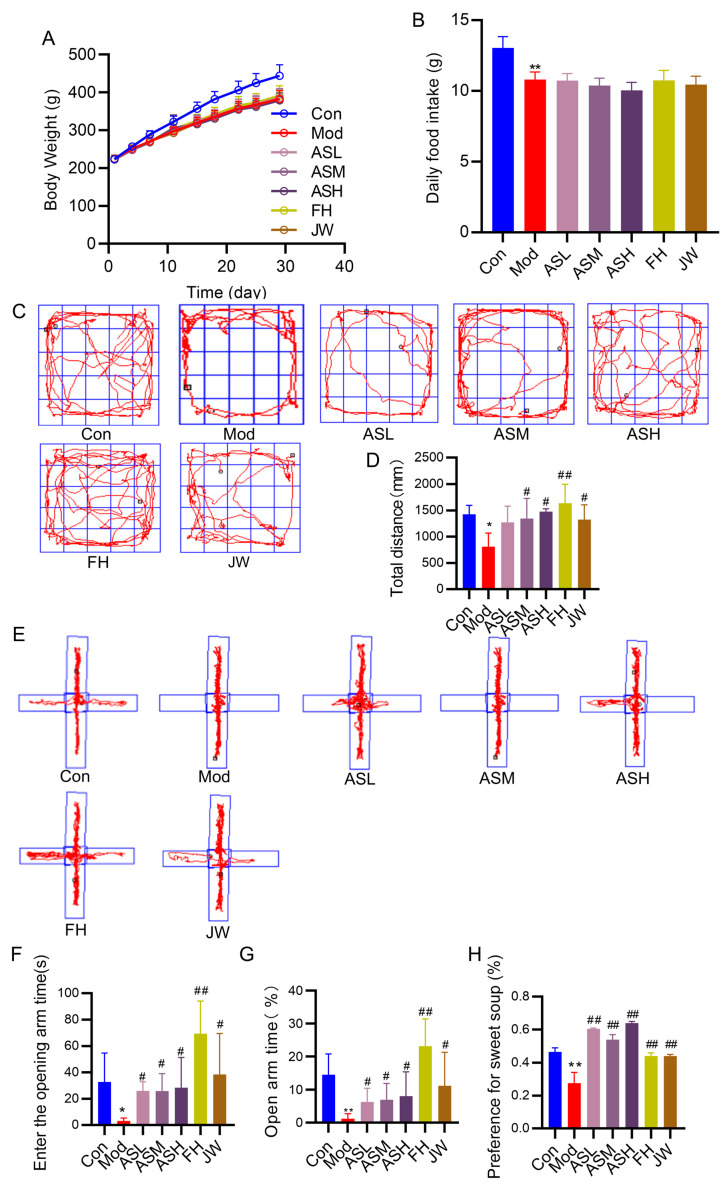
ABOL alleviates anxiety-like symptoms in anxious rats. (**A**) Rat body weight change curve; (**B**) Daily food intake; (**C**) Open field trajectory diagram; (**D**) Total distance traveled in open field; (**E**) Elevated plus maze trajectory diagram; (**F**) Time spent in open arms; (**G**) Distance traveled in central area of elevated plus maze; (**H**) Sucrose preference ratio. (*n* = 7). * *p* < 0.05 and ** *p* < 0.01 vs. CON; ^#^ *p* < 0.05 and ^##^ *p* < 0.01 vs. MOD.

This study aimed to evaluate the potential anxiety-inducing effects of chronic restraint stress (CRS) on anxiety-like behaviours in rats, as well as the potential ameliorative effects of ABOL on such behaviours. The body weight and food intake of the rats were monitored throughout the experiment. The results showed that, following CRS modelling, body weight gain was significantly inhibited and food intake decreased markedly in the model group. There were no statistically significant differences in body weight or food intake between the model group and the administration groups (ABOL, traditional Chinese medicine positive control, and western medicine positive control). These findings suggest that neither ABOL nor the positive control drugs had a significant impact on body weight gain or food consumption in rats (Figure 1A,B).

Subsequent behavioural tests further verified the anxiolytic efficacy of ABOL. In the Open Field Test (Figure 1C,D), spontaneous locomotor activity was significantly reduced in the model group and the movement trajectory exhibited typical thigmotaxis behaviour; the rats almost completely avoided the central open area, merely moving along the surrounding walls. This indicates evident anxiety- and depression-like states. Treatment with ABOL, traditional Chinese medicine (Jiuwei Zhenxin Granules) and western medicine (Fluoxetine Hydrochloride) significantly improved spontaneous locomotor activity and exploratory behaviours, effectively antagonising anxiety-induced abnormal behaviours. However, ABOL was less effective than the two positive control groups in increasing locomotor activity.

In the Elevated Plus Maze (EPM) test (Figure 1E,F), the number of entries into the open arms decreased significantly in the model group, with rats mainly confining themselves to the closed arms. This indicated a marked avoidance of exposed open areas and heightened vigilance, suggesting anxiety-like behaviours had worsened. ABOL administration effectively alleviated these avoidance behaviours, remarkably restoring the impaired exploration-avoidance balance in the anxious model rats. Although the ameliorative effect of ABOL was inferior to that of the two positive control groups, it still exhibited favourable anxiolytic potential.

The results of the Sucrose Preference Test (SPT) revealed that the sucrose preference rate was significantly reduced in the model group (Figure 1H), indicating obvious anhedonia. ABOL intervention effectively increased the sucrose preference rate of model rats, with a more significant improvement than the two positive control groups. This suggests that ABOL can effectively counteract the anhedonia induced by chronic restraint stress. It is speculated that the anxiolytic and potential antidepressant effects of ABOL may be mediated by the regulation of reward circuit mechanisms, such as the dopamine pathway.

In conclusion, ABOL can effectively enhance spontaneous locomotor activity and restore the normal balance between exploration and avoidance, as well as sucrose preference, in anxiety model rats, thereby exerting a definite anxiolytic effect.

### 2.2. Effects of ABOL on Neurotransmitter and HPA Axis Hormone Levels

**Figure 2:**
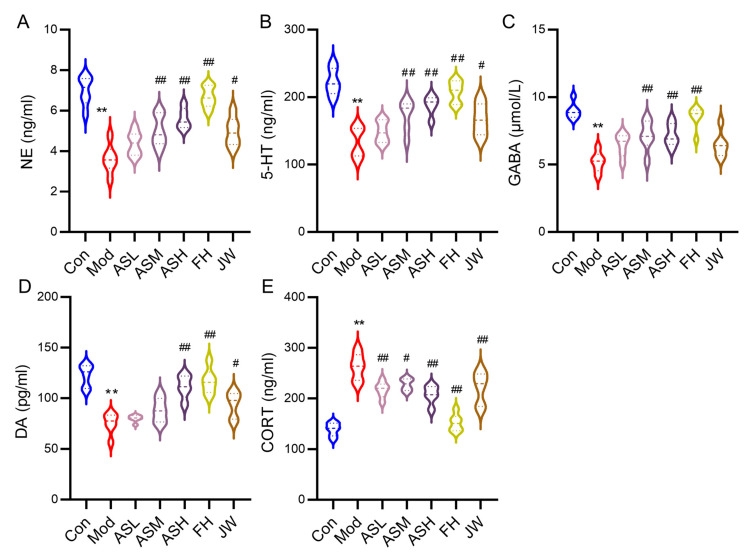
ABOL significantly improves neurotransmitter and HPA axis hormone levels in anxious rats. (**A**) NE content (ng/mL); (**B**) 5-HT content (ng/mL); (**C**) GABA content (μmol/L); (**D**) DA content (pg/mL); (**E**) CORT (ng/mL). (*n* = 6). ** *p* < 0.01 vs. CON; ^#^ *p* < 0.05 and ^##^ *p* < 0.01 vs. MOD. NE, Norepinephrine; 5-HT, 5-Hydroxytryptamine; GABA, γ-Aminobutyric Acid; DA, Dopamine; CORT, Corticosterone.

Anxiety has been demonstrated to induce dysregulation of neurotransmitters and HPA axis hormones. To investigate the regulatory effects of ABOL on these substances, the levels of NE, 5-HT, GABA, DA and CORT in the serum were measured using ELISA (Figure 2). The study revealed that, compared with the blank control group, the model group showed a significant decrease in neurotransmitter levels (NE, 5-HT, GABA and DA) and an increased CORT level. Following ABOL administration, NE, 5-HT, GABA and DA levels increased, while CORT levels decreased. This pattern of changes was consistent with that observed in the positive control groups, with the magnitude of the regulatory effect being intermediate between the fluoxetine hydrochloride group and the Jiuwei Zhenxin granules group. These results demonstrate the efficacy of ABOL in treating anxiety by modulating the balance of neurotransmitters and HPA axis hormones.

### 2.3. ABOL Improves Hippocampal Tissue Morphology in Anxiety-Prone Rats

In order to investigate the effects of ABOL on the hippocampal tissue of rats experiencing chronic restraint stress (CRS)-induced anxiety, histological staining and morphological observation of the hippocampus were performed.

The results showed that, in the blank control group, the neurons in the CA3 region of the hippocampus and the dentate gyrus (DG) exhibited intact structural morphology, with regular nuclei and clear nucleoli. No obvious pathological injuries, such as tissue edema, nuclear pyknosis or vacuolation, were observed.

**Figure 3:**
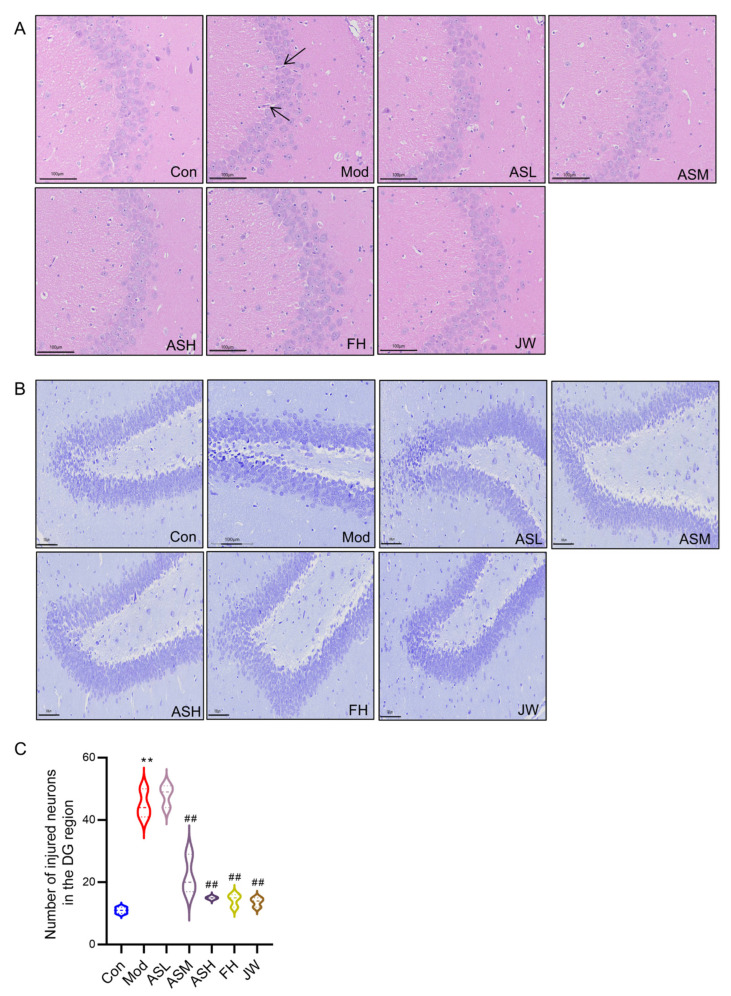
ABOL Reduces Neuronal Damage in the Hippocampus of Anxious Rats. (**A**) Hematoxylin and eosin staining of hippocampal CA3 region; (**B**) Nissl staining of hippocampal DG region; (**C**) Counted necrotic neurons in DG region. (*n* = 5); ** *p* < 0.01 vs. CON; ^##^ *p* < 0.01 vs. MOD. Black arrows in hippocampal tissue indicate neurite shrinkage. CA3, Cornu Ammonis 3; DG, Dentate Gyrus.

In contrast, the model group exhibited severe hippocampal neuronal damage, characterised by reduced neuronal count, somatic atrophy and extensive vacuolation. Following ABOL intervention, the aforementioned pathological injuries in the hippocampus of anxious rats were significantly alleviated, with evident reversal of neuronal atrophy. The neuroprotective effect of ABOL was comparable to that observed in the positive control group (Figure 3A,B).

Quantitative analysis of necrotic neurons in the DG region of the hippocampus further verified that ABOL treatment markedly decreased the number of necrotic neurons (Figure 3C).

In conclusion, ABOL can effectively alleviate anxiety-like behaviours in rats by reducing hippocampal neuronal damage.

### 2.4. ABOL Improves Colon Morphology and Maintains Intestinal Mucosal Integrity in Anxious Rats

**Figure 4:**
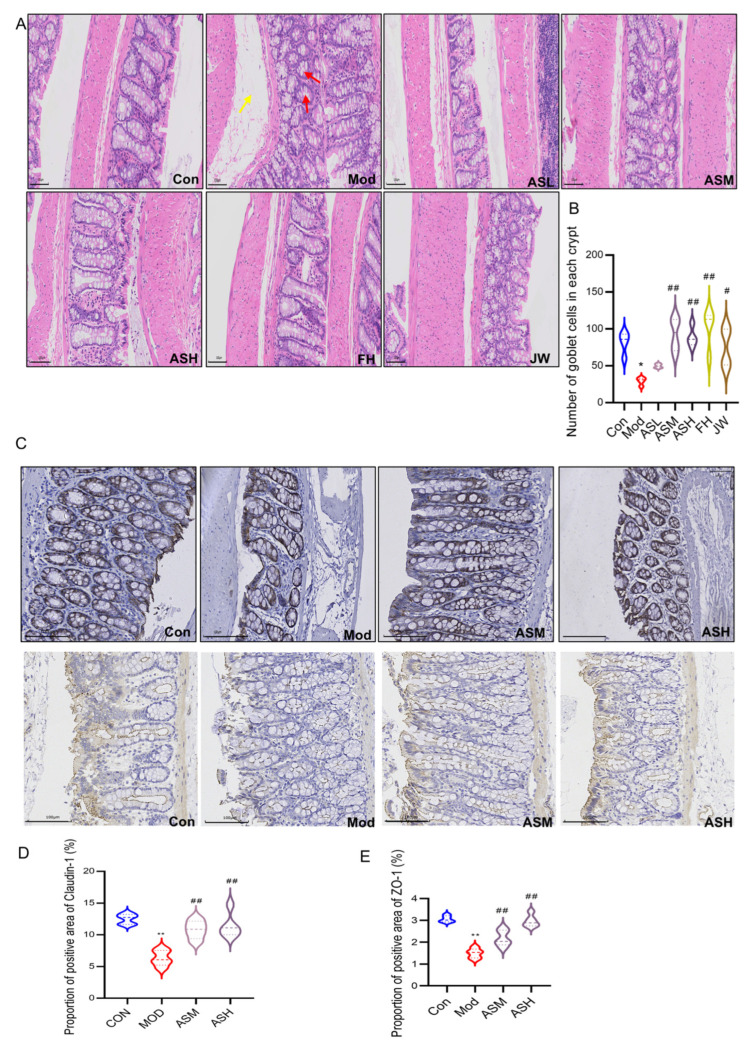
ABOL Improves Colonic Morphology and Maintains Intestinal Mucosal Integrity in Anxious Rats. (**A**) Colon HE staining; (**B**) Count of individual crypt goblet cells; (**C**) Positive expression of Claudin-1 and ZO-1 proteins in rat colon; (**D**) Percentage of positive area of Claudin-1 protein, (*n* = 5); (**E**) Percentage of positive area of ZO1 protein, (*n* = 5); Red arrows indicate submucosal edema, and yellow arrows represent altered crypt morphology. * *p* < 0.05, ** *p* < 0.01 vs. control group; ^#^ *p* < 0.05 and ^##^ *p* < 0.01 vs. MOD group. ZO-1, Zonula Occludens-1.

It has been demonstrated that anxiety induces structural damage to the colon and impairs the integrity of the intestinal barrier. To evaluate the protective effect of ABOL on colonic tissue, a histological analysis of rat colon samples was performed using haematoxylin and eosin (H&E) staining (Figure 4A). The results showed that the control group exhibited intact colonic mucosa, clearly visible goblet cells and regularly arranged crypts. In contrast, the model group displayed mucosal disruption, disorganised tissue structure, crypt atrophy and mild submucosal edema. Notably, ABOL treatment effectively reversed these pathological changes, restoring the colonic histological morphology to a state close to normal. The therapeutic efficacy of ABOL was comparable to that of the positive control groups.

Quantification of goblet cells per crypt (Figure 4B) revealed a statistically significant decrease in the model group compared to the control group, a difference that was significantly reversed following ABOL intervention. This finding confirms ABOL’s capacity to restore colonic mucosal architecture. Given the well-established correlation between anxiety and intestinal hyperpermeability, immunohistochemistry was employed to investigate the expression levels of key tight junction proteins (Claudin-1 and ZO-1) (Figure 4C). The expression of these tight junction proteins was significantly downregulated in the model group, indicating intestinal barrier leakage and impaired barrier function. In contrast, ABOL administration significantly increased the expression of these proteins, thereby restoring intestinal barrier integrity and reducing permeability.

Taken together, these results suggest that ABOL exerts a protective effect on the intestinal barrier by maintaining colonic mucosal structure and enhancing epithelial tight junctions, potentially underpinning its anti-anxiety activity.

### 2.5. ABOL Reduces Inflammatory Mediators and Oxidative Stress Levels in CRS-Induced Rats

**Figure 5:**
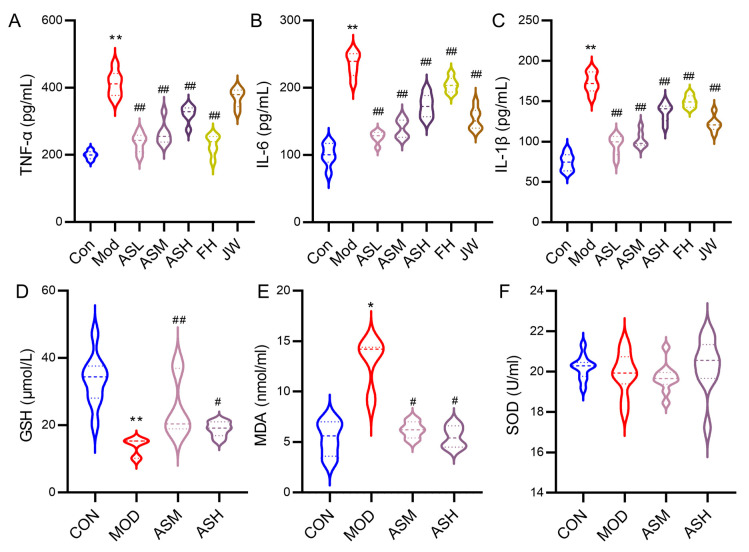
ABOL Improves Anxiety in Rats via Anti-inflammatory and Antioxidant Pathways. (**A**) Serum TNF-α; (**B**) Serum IL-6; (**C**) Serum IL-1β; (**D**) Serum GSH; (**E**) Serum MDA; (**F**) Serum SOD. (*n* = 5). * *p* < 0.05 and ** *p* < 0.01 vs. CON; ^#^ *p* < 0.05 and ^##^ *p* < 0.01 vs. MOD. TNF-α, Tumor Necrosis Factor-alpha; IL-6, Interleukin-6; L-1β, Interleukin-1 beta; GSH, Glutathione; MDA, Malondialdehyde; SOD, Superoxide Dismutase.

In light of the well-documented correlation between anxiety and inflammation and oxidative stress dysregulation, this study aimed to explore the therapeutic potential of ABOL. This was achieved by quantifying relevant serum markers, including tumour necrosis factor-α (TNF-α), interleukin-6 (IL-6), interleukin-1β (IL-1β), malondialdehyde (MDA), glutathione (GSH) and superoxide dismutase (SOD) (Figure 5).

Compared with the control group, rats in the model group exhibited significantly elevated serum levels of TNF-α, IL-6 and IL-1β, accompanied by significantly reduced GSH content. In contrast, SOD activity remained unchanged in the model group relative to the control group.

Notably, ABOL administration effectively reversed these abnormal changes, restoring the serum levels of inflammatory mediators and oxidative stress markers to normal ranges.

Taken together, these results imply that alleviating inflammation and oxidative stress may be a key mechanism underlying the anxiolytic effects of ABOL.

### 2.6. ABOL Alleviates Anxiety Induced by Chronic Stress Through Glycerophospholipid and Sphingolipid Metabolism

Pharmacodynamic studies have demonstrated that ABOL has an ameliorative effect on anxiety induced by chronic restraint stress (CRS). To elucidate the underlying metabolic mechanisms, a metabolomic analysis was conducted on rat plasma, and the key pathway-related genes were validated using RT-qPCR.

**Figure 6:**
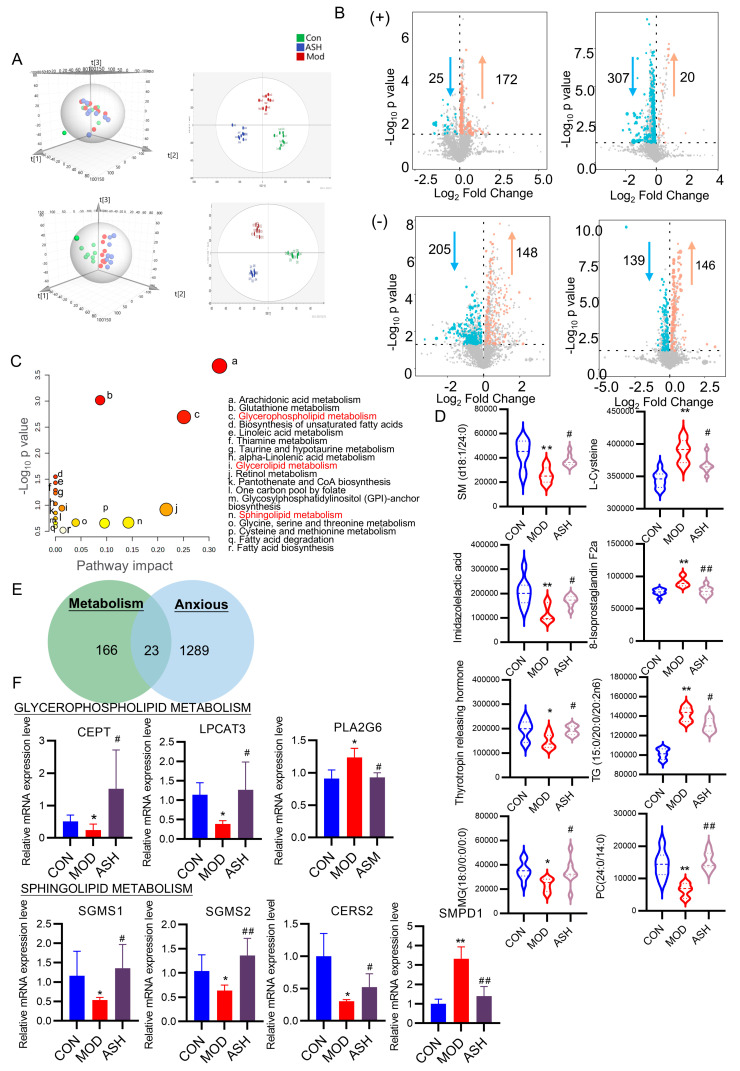
ABOL alleviates CRS-induced anxiety via glycerophospholipid and sphingolipid metabolism. (**A**) PCA 3D plots and OPLS-DA scoreplots for positive and negative patterns; (**B**) Metabolic volcano plot for positive and negative patterns; (**C**) Metabolic pathway enrichment diagram of ASH in alleviating anxiety; (**D**) Eight significantly altered metabolites regulated by ASH (*n* = 8); (**E**) VENN diagram of eight significantly altered metabolites and anxiety-related proteins; (**F**) Expression levels of key metabolic genes involved in glycerophospholipid and sphingolipid metabolism in brain tissue (*n* = 5). * *p* < 0.05 and ** *p* < 0.01 vs. CON; ^#^ *p* < 0.05 and ^##^ *p* < 0.01 vs. MOD. PCA, Principal Component Analysis; OPLS-DA, Orthogonal Partial Least Squares Discriminant Analysis.

Principal component analysis (PCA) and orthogonal projection to latent structures-discriminant analysis (OPLS-DA) 3D score plots (Figure 6A) revealed good intra-group clustering of samples, indicating low individual variation and high similarity among rats within the same group. Concurrently, distinct separation was observed between the control, model and ABOL groups, confirming the successful establishment of the CRS model and the presence of significant metabolic disturbances in model rats. ABOL treatment was also found to markedly alter metabolic profiles.

Screening employed VIP values from SIMCA, fold change (FC) and *p*-values (criteria: *p* < 0.05, FC ≥ 1 and VIP > 1). This was followed by analysis via the bioinformatics platform, which generated metabolite volcano plots (Figure 6B). In positive ion mode, 172 metabolites were found to be up-regulated and 25 down-regulated in the Model group compared to the Control group (MOD-CON), while 307 metabolites were down-regulated and 20 metabolites were up-regulated in the ABOL group compared to the Model group (ASH-MOD). In negative ion mode, MOD-CON exhibited 148 up-regulated and 205 down-regulated metabolites, whereas ASH-MOD displayed 146 up-regulated and 139 down-regulated metabolites. Combining both modes resulted in the identification of 39 potential differential metabolites (Table 1). Pathway analysis using MetaboAnalyst indicated that the high-dose ABOL group primarily influenced glutathione metabolism, arachidonic acid metabolism, glycerophospholipid metabolism and sphingolipid metabolism (Figure 6C). Subsequent analysis of the 39 metabolites identified eight that were significantly affected by ABOL (Figure 6D). Using the HMDB and Uniprot databases, 189 proteins associated with the eight metabolites and 1289 disease-related proteins were identified. Of these, 23 were linked to anxiety disorder (Figure 6E), which highlights the importance of these metabolites in ABOL’s anti-anxiety mechanism.

Visualisation of significant differential metabolites within metabolic pathways suggested that SM (d18:1/24:0) and PC (24:0/14:0) might play pivotal roles. Consequently, RT-qPCR validation was performed on genes that regulate the synthesis and metabolism of sphingomyelin and phosphatidylcholine within the sphingolipid and glycerophospholipid pathways (Figure 6F). The genes examined were sphingomyelin synthase 1 (SGMS1), sphingomyelin synthase 2 (SGMS2), sphingomyelin phosphodiesterase 1 (SMPD1), ceramide synthase 2 (CERS2), choline/ethanolamine phosphotransferase (CEPT), lysophosphatidylcholine acyltransferase 3 (LPCAT3) and phospholipase A2 (PLA2G6). In the sphingolipid metabolism pathway, SGMS1, SGMS2 and CERS2 expression levels were downregulated, while SMPD1 expression was upregulated in the model group. ABOL reversed these abnormal changes by upregulating SGMS1, SGMS2 and CERS2 expression and inhibiting SMPD1 expression. In the glycerophospholipid metabolism pathway, the model group exhibited decreased CEPT and LPCAT3 expression, as well as elevated PLA2G6 expression. ABOL increased CEPT and LPCAT3 levels and suppressed PLA2G6 expression. Overall, ABOL effectively reversed the aberrant expression of key genes involved in both the sphingolipid and glycerophospholipid metabolic pathways. This regulatory network appears to promote the synthesis of sphingomyelin and phosphatidylcholine whilst reducing their conversion to downstream products. Given the essential nature of sphingomyelin and phosphatidylcholine in cell membrane composition, the present findings suggest that ABOL may alleviate anxiety by modulating sphingolipid and glycerophospholipid metabolism. This, in turn, may result in the regulation of cell membrane permeability and enhancement of membrane signal transduction.

### 2.7. Modulating Effects of ABOL on Gut Microbiota in Rats with Chronic Restraint Stress-Induced Anxiety

**Figure 7:**
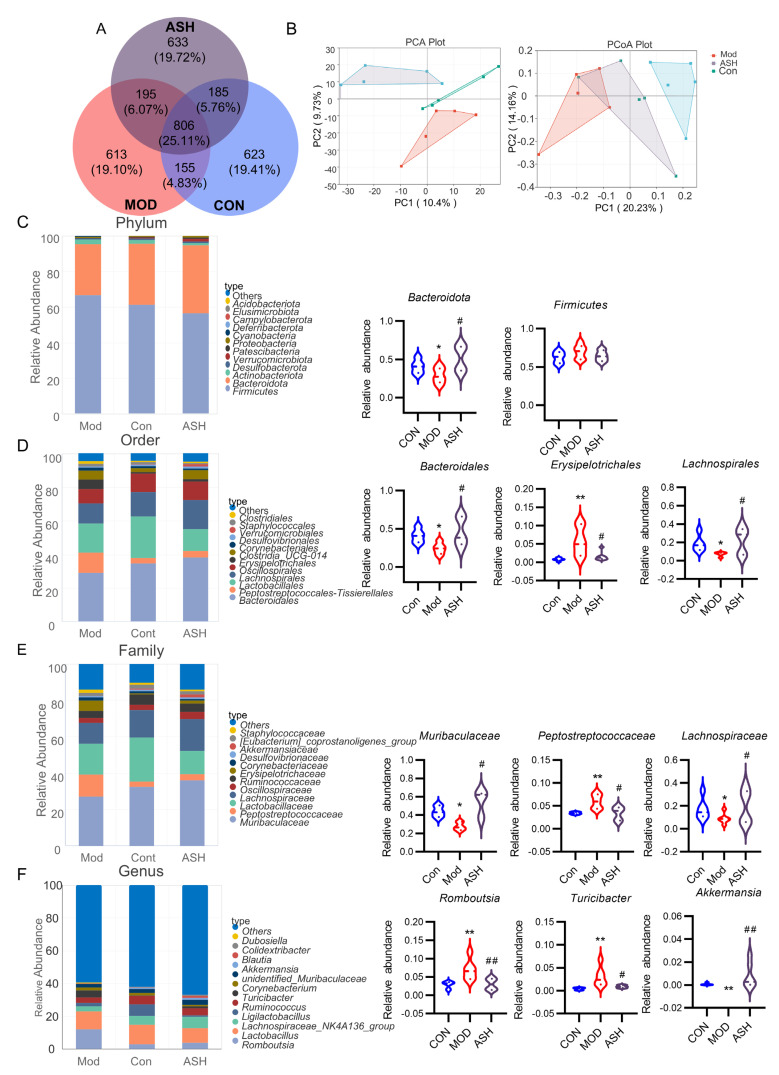
ABOL Reshapes the Gut Microbiota of Anxiety Rats. (**A**) Venn diagram showing OTU distribution differences among CON, MOD, and ASH groups; (**B**) PCA analysis and PCoA analysis; (**C**) Phylum-level abundance bar chart and corresponding violin plots of differentially abundant bacteria; (**D**) Family-level abundance bar chart and corresponding differential bacteria violin plot; (**E**) Order-level abundance bar chart and corresponding differential bacteria violin plot; (**F**) Genus-level abundance bar chart and corresponding differential bacteria violin plot; (*n* = 6). * *p* < 0.05 and ** *p* < 0.01 vs. CON; ^#^ *p* < 0.05 and ^##^ *p* < 0.01 vs. MOD. OTU, Operational Taxonomic Unit; PCoA, Principal Coordinate Analysis.

This study investigates the effect of ABOL on the gut microbiota of anxious rats. This study uses 16S rRNA sequencing to analyse the impact of ABOL on the microbiota. A Venn diagram illustrates the 806 OTUs common to all groups, indicating the presence of a stable core microbiota. Furthermore, the results showed that the ABOL intervention restored the gut microbiota in anxious rats (Figure 7A).

Alpha and beta diversity are comprehensive metrics for assessing microbial diversity and dissimilarity. The Chao1 index was used to estimate species richness and the Shannon index to evaluate community diversity. The results showed no statistically significant differences in the Chao1 and Shannon indices between the CON, MOD and ASH groups. Principal coordinate analysis (PCoA) was performed to analyse beta diversity (Figure 7B). The results indicated that the composition of the gut microbiota was altered in the MOD group, with changes in species evenness and richness. Conversely, ABOL treatment demonstrated a restorative effect.

Bar plots of species abundance displayed the relative abundances of the top 12 gut microbial taxonomic units. At the phylum level (Figure 7C), the most dominant gut microbes in each group were Firmicutes and Bacteroidota. Compared with the blank control (CON) group, the MOD group exhibited an increased abundance of Firmicutes and a decreased relative abundance of Bacteroidota. ABOL intervention ameliorated this phylum-level imbalance.

At the order level (Figure 7D), the MOD group showed a decreased relative abundance of *Bacteroidales* and an increased relative abundance of *Erysipelotrichales* and *Lachnospirales*, compared with the CON group. ABOL intervention shifted the order-level microbiota composition back towards the normal state. At the family level (Figure 7E), the MOD group exhibited decreased relative abundances of *Muribaculaceae* and *Lachnospiraceae* compared with the CON group, and an increased relative abundance of *Peptostreptococcaceae*. ABOL intervention corrected this family-level dysbiosis. At the genus level (Figure 7F), the MOD group exhibited significantly higher abundances of *Romboutsia* and *Turicibacter*, as well as significantly lower abundances of *Akkermansia*. ABOL intervention effectively ameliorated gut microbial dysbiosis.

In summary, ABOL modulates the structure of the gut microbiota across multiple taxonomic levels. The intervention reversed the anxiety-induced imbalance in the *Firmicutes*/*Bacteroidota* ratio and regulated the abundance of key taxa, such as *Erysipelotrichales* and *Lachnospirales*. Furthermore, ABOL has been demonstrated to increase the abundance of beneficial genera, such as *Akkermansia* and Clostridia_UCG_014, while concomitantly decreasing the abundance of potentially harmful genera, such as *Romboutsia* and *Turicibacter*. This ultimately restores gut microbiota homeostasis. This study suggests that modulating key gut microbiota is an important mechanism through which ABOL exerts its anxiolytic effects.

**Figure 8:**
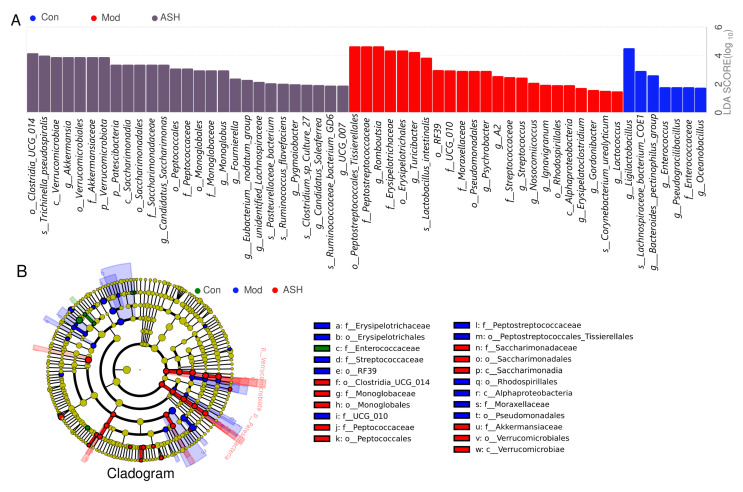
ABOL may induce specific microbial enrichment and differential interactions between bacteria and metabolites. (**A**) Linear Discriminant Analysis (LDA) scores for differentially expressed bacteria; (**B**) Phylogenetic tree of differentially expressed bacteria.

The LefSe analysis was performed on the CON, MOD, and ASH groups (Figure 8A,B) to identify taxa that were enriched in the different populations. The MOD group was characterised primarily by enrichments in families such as *Erysipelotrichaceae* and *Peptococcaceae*, while the ASH group was predominantly enriched in taxa including Clostridia_UCG_014 and *Akkermansia*. These findings suggest that ABOL contributes to the restoration of the gut microbial community. The study demonstrates that ABOL alleviates anxiety, potentially by regulating key microbial groups, including *Akkermansiaceae*, Clostridia_UCG_014, and *Verrucomicrobiae*.

## 3. Discussion

ABOL is a traditional Chinese herbal remedy that is widely used in clinical practice, primarily for alleviating symptoms related to insomnia and neurasthenia. As anxiety disorders and insomnia are closely comorbid and share pathological mechanisms, it is hypothesised that ABOL has therapeutic potential for anxiety disorders. The gut–brain axis plays a core regulatory role in the pathogenesis and progression of anxiety disorders. The present study employed a chronic restraint stress (CRS)-induced anxiety-like rat model to systematically evaluate the ameliorative effects of ABOL on anxiety-like behaviours, and analyse its regulatory role in gut microbiota structure and metabolite imbalance. This would provide experimental evidence for elucidating the anxiolytic mechanism of ABOL. Fluoxetine hydrochloride capsules and Jiuwei Zhenxin Granules were selected as positive controls for pharmacodynamic evaluation. Fluoxetine, a selective serotonin reuptake inhibitor (SSRI), enhances serotonergic neurotransmission by potently inhibiting neuronal serotonin reuptake [35]. Jiuwei Zhenxin Granules has been shown to have favourable therapeutic effects on generalised anxiety disorder. Therefore, it was chosen as a positive control together with fluoxetine. The gradient doses used in this study were established by referring to the published administration regimen of Anshen Bunao Oral Liquid for its neuroprotective effects on cognitive dysfunction in Alzheimer’s disease model rats [30]. Combined with behavioral evaluation, detection of neurotransmitter and inflammatory cytokine levels, as well as PCA multivariate analysis results of metabolomics and microbiomics, comprehensive assessment confirmed that the high-dose group achieved the optimal intervention effect. Accordingly, subsequent research in this study focused on the high-dose group.

Anxiety-like behaviour was induced in rats via chronic restraint stress (CRS), and behavioural alterations were subsequently assessed using the open field test (OFT), elevated plus maze (EPM) and sucrose preference test (SPT). Behavioural analysis revealed that the model rats displayed reduced sucrose preference in the SPT, indicating anhedonia, as well as markedly decreased locomotor and exploratory activity in both the EPM and OFT. These consistent behavioural abnormalities confirmed the successful establishment of an anxiety-like state and verified that chronic restraint stress is a reliable approach for constructing a rat anxiety model. Notably, ABOL was found to have obvious ameliorative effects on CRS-induced anxiety-like behaviours, with therapeutic efficacy comparable to the positive controls. In the open field test (OFT), ABOL’s anxiolytic effect was weaker than fluoxetine’s, whereas ABOL’s effect in the sucrose preference test (SPT) was superior. This divergent behavioural performance may be explained by their distinct pharmacological mechanisms. Fluoxetine acts mainly by selectively inhibiting 5-HT reuptake, a relatively singular mechanism. By contrast, ABOL, a traditional Chinese medicinal compound, concurrently elevates the levels of 5-HT, DA and NE. Since DA is a key neurotransmitter that regulates reward response and sweet taste preference, the multiple components of ABOL can enhance DA synthesis and release synergistically, robustly activate the brain reward circuit and improve sucrose preference more effectively than fluoxetine, which has a single therapeutic target.

As a classic antidepressant, fluoxetine exhibits more stable and pronounced anxiolytic effects. The OFT is a well-established method for evaluating anxiety-like behaviour in experimental animals; therefore, the consistent efficacy of fluoxetine results in improved performance in this test, which accounts for its superior anxiolytic effect compared to ABOL in the OFT. ELISA results showed that ABOL increased peripheral blood levels of the neurotransmitters 5-hydroxytryptamine (5-HT), γ-aminobutyric acid (GABA), dopamine (DA) and norepinephrine (NE), while decreasing corticosterone (CORT) levels. Consistent with previous studies [36,37,38], these findings suggest that ABOL exerts its anxiolytic effects by regulating the levels of 5-HT, NE, GABA, DA and CORT in rats with CRS-induced anxiety.

Neuroinflammation and oxidative stress are key factors in anxiety, with their levels strongly correlating with anxiety severity [11,39]. We found that ABOL reduced the serum levels of the pro-inflammatory cytokines TNF-α, IL-6 and IL-1β in rats with chronic restraint stress-induced anxiety. This demonstrates its capacity to lower peripheral inflammatory cytokine levels. Furthermore, ABOL increased serum glutathione (GSH) levels and decreased malondialdehyde (MDA) levels, but had no significant effect on superoxide dismutase (SOD) levels. The lack of effect on SOD may be due to variations in SOD activity among different tissues and in local oxidative stress levels, as well as other related factors. These findings suggest that ABOL exerts significant antioxidant effects and can reduce oxidative stress in rats with anxiety. Alongside the changes in neurotransmitter levels consistent with previous research [40,41], these results suggest that the anxiolytic mechanism of ABOL may involve the suppression of inflammation and oxidative stress.

Histopathology is a straightforward method of observing morphological changes in tissues and cells to determine disease severity at the cellular level. The hippocampus is one of the brain regions where neural stem cells reside and neurogenesis can be sustained. As a key target of stress regulation, the hippocampus is closely associated with the development of psychological disorders such as anxiety and depression [14]. Haematoxylin-eosin (HE) and Nissl staining of hippocampal tissue revealed that chronic restraint stress (CRS) led to a slight decrease in the number of neurons, accompanied by neuronal shrinkage, deeper staining and abnormal morphology. Following ABOL intervention, however, hippocampal pathology improved significantly and neuronal damage was effectively alleviated. Chronic exposure to stress increases the risk of psychological disorders such as anxiety and depression, and is also associated with impaired intestinal barrier function [42]. HE staining of intestinal tissue showed that the intestinal mucosa of model rats was damaged, exhibiting submucosal edema, irregular crypt structures and a significant reduction in goblet cells. Following ABOL treatment, mucosal damage was effectively alleviated and the mucosal layer returned to normal. These results demonstrate that ABOL possesses anxiolytic properties and alleviates hippocampal neuronal damage while maintaining intestinal mucosal integrity. Immunohistochemistry (IHC) further confirmed the impact of intestinal mucosal integrity and permeability on anxiety. Compared with the blank control group, the expression levels of ZO-1 and Claudin-1 were significantly lower in the model group. However, ABOL treatment increased the expression of these two tight junction proteins. Taken together, these findings suggest that ABOL mitigates anxiety by reducing hippocampal neuronal damage and preserving intestinal mucosal integrity.

In an untargeted metabolomics analysis of plasma, the enrichment analysis of significantly reversed differential metabolites revealed that ABOL primarily impacts glycerophospholipid and sphingolipid metabolic pathways in the treatment of anxiety. The central nervous system (CNS) consists of three types of lipid: phospholipids, sphingolipids, and cholesterol. The most abundant glycerophospholipid in eukaryotic cell membranes is phosphatidylcholine (PC), accounting for 40–50% of total membrane composition. As a key component of neuronal cell membranes, PC directly regulates membrane fluidity. Previous studies have shown that PC 36:4, an alkylacyl phosphatidylcholine, is negatively correlated with depressive phenotypes and may serve as a potential biomarker [43]. Our research revealed that chronic restraint stress (CRS) leads to a decrease in PC (24:0/14:0) levels. PC (24:0/14:0) is a phospholipid species comprising long- and short-chain saturated fatty acids linked to a glycerol backbone. It plays a crucial role in maintaining the structural stability and functional integrity of neural membranes. A decrease in phospholipids such as this may be a key factor in abnormal neurotransmission and emotional regulation disorders. Further research is needed on different types of phosphatidylcholine (PC) to explore their impact on various mental illnesses. Sphingolipid metabolism is also a major metabolic pathway through which ABOL influences anxiety. Studies assessing the association between plasma metabolites and the risk of neurodegenerative diseases have found that all plasma metabolites involved in sphingolipid metabolism can influence mental health conditions [44]. Additionally, studies have shown that reduced levels of SM (40:2) and SM (41:1) promote anxiety-like behaviours in rats fed a high-sugar diet [45], and that increased acid sphingomyelinase (ASM) activity leads to decreased sphingolipid levels and elevated ceramide concentrations in rodent models of major depressive disorder (MDD) [12]. In the present study, SM (d18:1/24:0) levels were significantly lower in the model group than in the control group, while ABOL administration increased them. This trend is similar to the reduction in sphingolipid levels observed in depression. To verify the expression changes of phosphatidylcholine (PC) and sphingomyelin (SM), we performed a reverse transcription quantitative polymerase chain reaction (RT-qPCR) on their respective regulatory genes. Sphingosine is converted to ceramide by ceramide synthase (CERS), and ceramide is further converted to SM by sphingomyelin synthase. In this study, we detected increased expression of CERS and decreased expression of SGMS1 and SGMS2, leading to elevated ceramide concentrations and reduced SM levels. Similarly, choline/ethanolamine phosphotransferase (CEPT) and lysophosphatidylcholine acyl-transferase 3 (LPCAT3) are synthases involved in PC biosynthesis, whereas phospholipase A2 group VI (PLA2G6) hydrolyses PC into lysophosphatidylcholine or arachidonic acid, thereby activating neuroinflammation or disrupting cell membrane function. Increased PLA2G6 expression has also been observed in anxiety models [46]. This study detected decreased expression of CEPT and LPCAT3, as well as increased expression of PLA2G6, in the model group. Administering ABOL reversed the expression of these genes, thereby reducing PC hydrolysis and promoting SM synthesis. Taken together, these findings suggest that ABOL modulates lipid metabolism via the glycerophospholipid and sphingolipid metabolic pathways. This modulation maintains cell membrane integrity and permeability, alleviates neuroinflammation and ultimately exerts a therapeutic effect on anxiety.

The gut microbiota plays a crucial role in various physiological processes, and dysbiosis of the microbiota is associated with a variety of neurological disorders. It can modulate the production of neurotransmitters and their precursors, as well as secrete and upregulate essential proteins and metabolites involved in the release of neuropeptides and gut hormones. This regulates intestinal barrier function [13,47]. In the analysis of the effects of ABOL on the gut microbiota, multivariate statistical analyses (principal component analysis, PCA; and principal coordinate analysis, PCoA) demonstrated that the animal model had been successfully established. The two core phyla of the gut microbial community are *Firmicutes* and *Bacteroidota*, which collectively constitute the main component of the gut microbiota. *Firmicutes* is the dominant phylum, exhibiting a high relative abundance. *Bacteroidota* synergistically maintains microecological balance with *Firmicutes*, and a decrease in its abundance is associated with an increased risk of inflammatory bowel disease (IBD) and autoimmune diseases. The *Firmicutes*/*Bacteroidota* ratio (F/B ratio) can serve as a biomarker of the organism’s pathological state. Studies have shown that patients with depression and anxiety have an increased abundance of *Firmicutes* and a decreased abundance of *Bacteroidota*, resulting in an elevated F/B ratio [48]. This imbalance is associated with inflammation, a phenomenon that we also observed in the model group. In the chronic unpredictable mild stress (CUMS)-induced anxiety model, the abundance of *Bacteroidota* and *Lachnospiraceae* were decreased following CUMS exposure [49,50]. *Muribaculaceae* can produce short-chain fatty acids (SCFAs), which have anti-inflammatory and neurotransmitter-modulating effects [51]. This is crucial for maintaining barrier integrity in the body. A decrease in these three bacterial taxa was also observed in our experiment, and administration of ABOL restored their abundances. At the genus level, we focused on *Romboutsia*, *Turicibacter*, and *Akkermansia*. High abundances of *Romboutsia* and *Turicibacter* have been observed in individuals with high trait anxiety and in animal models of anxiety [52,53]. Increased *Romboutsia* has also been reported in colitis models; however, studies on its association with mental illnesses are limited, and this genus may act as a pro-inflammatory pathogenic bacterium [54]. *Akkermansia* contributes to maintaining a healthy intestinal barrier, regulating immunity, inhibiting inflammation and being involved in various mental illnesses [55,56]. In our study, ABOL reversed the significant decrease in *Akkermansia* abundance observed in the model group. Taken together, these findings suggest that ABOL exerts therapeutic effects on anxiety by increasing the abundance of beneficial bacteria and decreasing that of harmful bacteria. Such regulation is associated with modulating neuroinflammation and maintaining intestinal barrier integrity.

The present study has several inherent limitations. Firstly, a dedicated dietary control group was not set up. This may affect our ability to interpret the intervention effect of ABOL. Secondly, the relatively small sample size for 16S rRNA sequencing (six replicates per group) may reduce the statistical power to identify differential microbial taxa. Thirdly, the lack of measurement of intestinal short-chain fatty acids limited our ability to elucidate the microbial metabolic functions underlying anxiety regulation in depth. Fourthly, correlation analyses between the gut microbiota and metabolomics revealed only associative relationships; targeted intervention studies are required to confirm whether specific microbial species and pivotal metabolites directly mediate the anxiolytic effect of ABOL. Fifthly, the regulatory mechanism of lipid metabolism pathways was inferred based solely on the expression levels of key mRNA. However, transcriptional changes do not always reflect protein abundance or enzymatic activity.

Future studies should therefore measure the expression and activity of core lipid metabolic enzymes to validate these molecular conclusions. In future research, we will increase the sample size for 16S rRNA sequencing, include a proper dietary control group and quantify intestinal short-chain fatty acids. We will also design combined intervention and verification experiments to clarify the causal relationships among characteristic microbiota, differential metabolites and anxiety phenotypes. Furthermore, we will supplement protein expression and enzymatic activity assays of key lipid metabolic enzymes at the post-transcriptional level, thereby providing more solid experimental evidence for the anxiolytic mechanism of ABOL.

## 4. Materials and Methods

### 4.1. Drugs and Reagents

ABOL (Batch No.: 2407214), provided by Jilin Aodong Yanbian Pharmaceutical Co., Ltd. (Dunhua, China) was purchased commercially. The formulation and standardized preparation process were in accordance with the requirements of the Chinese Pharmacopoeia [57]. The detailed preparation procedure and pharmacodynamic material basis are available in the Supplementary Material; Fluoxetine Hydrochloride Capsules (No.: 23363A), purchased from Lilly France (Fegersheim, France); Jiu Wei Zhen Xin Granule (No.: 08240212), manufactured by Beijing Beilu Pharmaceutical Co., Ltd. (Beijing, China); Malondialdehyde (MDA) assay kit, Superoxide Dismutase (SOD) assay kit, and Glutathione (GSH) assay kit were all purchased from Nanjing Jian cheng Bioengineering Institute (Nanjing, China); Rat Tumor Necrosis Factor-α (TNF-α) ELISA Kit (Cat. No.: ml002859), purchased from Shanghai Enzyme-linked Biotechnology Co., Ltd. (Shanghai, China); Rat Interleukin-6 (IL-6) ELISA Kit (Cat. No.: ml064292), purchased from Shanghai Enzyme-linked Biotechnology Co., Ltd.; Rat Interleukin-1β (IL-1β) ELISA Kit (Cat. No.: ml037359), purchased from Shanghai Enzyme-linked Biotechnology Co., Ltd.; Claudin-1 Antibody (Batch No.: GB12032); ZO-1 Antibody (Batch No.: GB151981);HRP-conjugated goat anti-rabbit IgG (Batch No.: GB21303); HRP-conjugated goat anti-mouse IgG (Batch No.: GB23301), All the above antibodies were obtained from Wuhan Servicebio Technology Co., Ltd. (Wuhan, China); 4% Paraformaldehyde Fixative (Batch No.: 230210J), produced by Guangzhou Jingxin Biotechnology Co., Ltd. (Guangzhou, China); Environmentally friendly dewaxing solution, Hematoxylin staining solution, Eosin staining solution, Hematoxylin differentiation solution, Hematoxylin bluing solution, and DAB chromogenic solution were purchased from Wuhan Servicebio Technology Co., Ltd.; Nissl Staining Solution (Batch No.: C0117), provided by Shanghai Beyotime Biotechnology Co., Ltd. (Shanghai, China); Sodium Citrate Antigen Retrieval Solution (Cat. No.: AR0024), purchased from BOSTER Biological Technology Co., Ltd. (Wuhan, China).

### 4.2. Experimental Animals

Seventy male Sprague-Dawley (SD) rats, aged 6–8 weeks and weighing 200 ± 20 g, were provided by the Guangdong Provincial Medical Experimental Animal Center. The Production License of Experimental Animals is SCXK (Yue) 2022–0002. The animal ethics approval number for this study is GDPULACSPF2022610. The animals were housed under controlled conditions at 18–23 °C and 45–55% relative humidity. The study protocol complied with the principles of animal protection, welfare, and ethics, and conformed to relevant national regulations, thereby permitting the experiment. Prior to the formal experiment, the SD rats were given free access to food and water and acclimatized for 3 days. They were then randomly assigned to seven groups (*n* = 10 per group) according to body weight: normal control group, model group, fluoxetine hydrochloride group (positive control), Jiuwei Zhenxin Granules group (positive control), ABOL low-dose group, ABOL middle-dose group, and ABOL high-dose group. The middle-dose (ASM) was based on the clinical adult daily intake of 20 mL (for a 60 kg individual) converted to a rat-equivalent dose using standard allometric scaling; the low-dose (ASL) and high-dose (ASH) groups were set at approximately 50% and 200% of this dose, respectively, to investigate the dose–response relationship [30].

### 4.3. Establishment and Treatment of the Anxiety Animal Model

**Figure 9:**
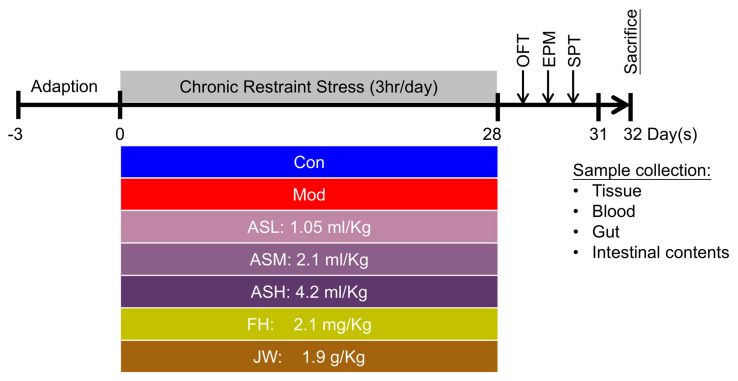
Experimental Protocol.

A rat model of anxiety disorder was established via chronic restraint stress. Rats were tightly restrained in restraint devices to restrict their free movement, with the openings kept open to ensure unobstructed breathing. The restraint treatment was performed for 3 h daily for 28 consecutive days [58]. The fluoxetine hydrochloride group and the Jiuwei Zhenxin Granules group received their respective drugs at the corresponding doses; the ABOL groups were intragastrically administered low, middle, or high doses of ABOL, while the normal control group and model group received an equal volume of distilled water. The administration lasted for 4 consecutive weeks (experimental protocol shown in Figure 9).

### 4.4. Behavioral Experiments

We conducted a series of behavioral tests, including the elevated plus maze test, open field test, and sucrose preference test, to evaluate the successful establishment of an anxiety model in rats.

#### 4.4.1. Elevated Plus Maze (EPM) Test

The EPM test is primarily used to assess anxiety-like behaviors in experimental rodents. The EPM apparatus consists of two opposite open arms and two opposite closed arms, arranged in a cross shape. The open arms are brightly lit with no shielding, while the closed arms are dim and secure. When rats are placed in the center of the maze, their behavioral parameters—including the time spent in the open and closed arms, the number of entries into each arm, and the distance traveled and stay time in the central area—can reflect their anxiety state. Rats with high anxiety levels tend to stay in the closed arms, whereas those with low anxiety levels spend more time exploring the open arms and enter them more frequently.

#### 4.4.2. Open Field Test (OFT)

The test was conducted in an open field box (50 cm × 50 cm × 50 cm) made of black material, in a quiet environment. Prior to each test, the open field box was cleaned and kept dry. The camera was adjusted to fully capture the entire open field area on the computer program, followed by setting the experimental parameters: a test duration of 5 min and a 1 s delay before timing (to allow the experimenter to withdraw their hand after placing the rat in the center of the box, avoiding false recognition by the system). By observing the movement trajectory and behavioral characteristics of rats in an open and unfamiliar environment, their anxiety state and motor ability were evaluated. A video tracking system was used to record and analyze the total distance traveled by the rats within 5 min during the OFT. Between consecutive tests, rat excreta were removed, and the experimental equipment was cleaned with 75% medical alcohol to eliminate residual odors or cues from previous rats that might affect subsequent experiments.

#### 4.4.3. Sucrose Preference Test (SPT)

The sucrose preference test is used to assess anhedonia—a diminished interest in rewarding stimuli, which is a core feature of emotional disorders—in rodents. The test consists of two phases: adaptation and testing. During the adaptation phase, two bottles of 1% sucrose solution were placed in each cage for 24 h. In the next 24 h, one bottle was replaced with pure water, and the positions of the two bottles were switched periodically to prevent side preference. Animals had free access to food and water during these two days. After adaptation, food and water were withheld for 24 h, followed by the test phase. Before testing, both bottles were weighed. Each rat was then presented with one bottle of 1% (*w*/*v*) sucrose solution and one bottle of pure water for 24 h. The positions of the bottles were switched during the test period. After 24 h, the bottles were removed and weighed again. The consumption of sucrose solution and pure water was recorded, and the total liquid consumption and sucrose preference percentage were calculated.

### 4.5. Sample Collection

After the completion of behavioral experiments, rats were fasted for 12 h with free access to water, then anesthetized with isoflurane. Blood samples were collected from the abdominal aorta, allowed to stand for 2 h, and then centrifuged at 3500 rpm (4 °C, 15 min). The supernatant was harvested and stored at −80 °C. Following blood collection, cardiac perfusion was performed with pre-cooled PBS. Rats were then decapitated to isolate the whole brain. For five rats per group, the whole brain was placed in 50 mL centrifuge tubes containing 4% paraformaldehyde and stored at 4 °C for more than 48 h. Subsequently, coronal sections were prepared through dehydration, clearing, and embedding procedures. The remaining brain tissues were dissected to separate the hippocampus, cerebral cortex, and other brain regions, which were then stored in sterile cryovials at −80 °C. After brain tissue dissection, the liver, colon, and colonic contents were harvested via anatomical dissection, quenched in liquid nitrogen, and stored at −80 °C.

### 4.6. Measurement of Neurotransmitter, Pro-Inflammatory Cytokine, Oxidative Stress and HPA Axis Hormone Levels

Rat serum was collected and assayed using enzyme-linked immunosorbent assay (ELISA) kits and oxidative stress detection kits. ELISA was employed to measure the peripheral blood levels of 4 neurotransmitters, 1 HPA axis hormone, and 3 pro-inflammatory cytokines: serotonin (5-HT), dopamine (DA), norepinephrine (NE), γ-aminobutyric acid (GABA), corticosterone (CORT), tumor necrosis factor-α (TNF-α), interleukin-6 (IL-6), and interleukin-1β (IL-1β). The contents of superoxide dismutase (SOD), malondialdehyde (MDA), and glutathione (GSH) in serum were determined using oxidative stress detection kits.

### 4.7. Histopathology and Immunohistochemistry

#### 4.7.1. Hematoxylin and Eosin (H&E) Staining

Whole brain tissues and colons fixed in 4% paraformaldehyde were subjected to routine dehydration, followed by paraffin embedding and sectioning. Brain tissue sections were prepared at a thickness of approximately 5–8 μm. After dewaxing with xylene, the sections were stained with H&E. Pathological observations of brain and colon tissues were performed using a multispectral tissue section analysis system, and images were captured.

#### 4.7.2. Nissl Staining

Paraffin-embedded sections were routinely deparaffinized, rehydrated, and washed sequentially in water. The sections were stained with methylene blue solution for 10 min, differentiated for 3 min, rinsed with distilled water and 95% ethanol, and finally mounted with neutral balsam. The number of necrotic neurons was observed and counted under a microscope.

#### 4.7.3. Immunohistochemistry (IHC)

Paraffin sections were routinely deparaffinized and rehydrated. After antigen retrieval and quenching of endogenous peroxidase activity, the sections were blocked with 3% BSA for 1 h, followed by incubation with primary antibodies overnight at 4 °C. After washing with PBS, the corresponding HRP-labeled secondary antibodies were applied. Color development was performed using DAB, followed by counterstaining with hematoxylin, differentiation, and bluing. The sections were mounted with neutral balsam. The expression of ZO-1 and Claudin-1 in the rat intestinal mucosa was detected, and the percentage of protein-positive staining area was analyzed using ImageJ (version 1.48v, National Institutes of Health, Bethesda, MD, USA) software.

### 4.8. Plasma Untargeted Metabolomics

Plasma samples stored at −80 °C were first thawed at 4 °C for 30 min and then completely thawed at room temperature. After thawing, 10 μL of plasma from each sample was pooled to prepare quality control (QC) samples; these QC samples were processed simultaneously with the experimental samples. For each sample, 200 μL of plasma was transferred to a 1.5 mL centrifuge tube, mixed with 600 μL methanol, and vortexed for 1 min. The mixture was centrifuged at 14,000 rpm for 15 min at 4 °C, and the supernatant was filtered through a 0.22 μm microporous membrane before instrumental analysis.

Chromatographic separation was performed using an Acquity UPLC BEH C18 column (2.1 mm × 100 mm, 1.7 μm). The mobile phase consisted of 0.1% formic acid in water (A) and acetonitrile (B). The flow rate was 0.3 mL/min, the column temperature was maintained at 40 °C, and the injection volume was 2 μL. The linear elution gradient was set as follows: 0–2 min, 99% A/1% B; 5 min, 75% A; 15 min, 50% A; 28 min, 40% A; 35–43 min, 100% B; 48–58 min, 99% A.

Mass spectrometric data were acquired within the range of 0.1–40 min. The ion source gas flow was 50 psi, and the curtain gas flow was 35 psi. The ion source temperature was 600 °C, the capillary ionization voltage was 5500 V, and the declustering voltage was 100 V. The mass scan range was set separately for the two modes: full scan TOF-MS from 100 to 1500 Da, and product ion scan from 50 to 1000 Da. The accumulation acquisition time was 0.25 s for TOF-MS and 0.1 s for product ion scanning. The collision energy was 5 V for full scan mode and 40 V ± 20 V for product ion mode. The minimum peak intensity was defined as 10% of the base peak intensity with a minimum peak width of 50 ppm. Up to six mass peaks were monitored simultaneously within the same retention time window, with an allowable retention time deviation of 0.05 min and a mass-to-charge ratio (*m*/*z*) tolerance of 50 ppm.

Raw mass spectrometry data were preprocessed using MarkerView software ((version 1.2.1, AB Sciex, Concord, ON, Canada). The preprocessed data were imported into SIMCA-P 13.0 for principal component analysis (PCA) and partial least squares discriminant analysis (PLS-DA). Differential metabolites were screened using the criteria of variable importance in projection (VIP) > 1 from the PLS-DA model and *p* < 0.05 by one-way ANOVA.

Tentative identification of differential metabolites was performed using PeakView software (version 1.2, AB Sciex, Concord, ON, Canada). Elemental compositions were predicted based on isotopic peak intensity ratios, and database matching was conducted against HMDB, PubChem, NIST, MassBank, and KEGG via the built-in ChemSpider service. Compound structures were elucidated by interpreting MS/MS fragment ions and rational bond cleavage patterns. Metabolites that were not endogenously present in blood or were identified as exogenous contaminants were excluded.

Metabolic pathway enrichment analysis of differential metabolites was conducted using MetaboAnalyst 6.0. Significantly enriched pathways were screened with the criteria of *p* < 0.05 and pathway impact value > 0.2.

### 4.9. 16S rRNA Sequencing and Bioinformatics Analysis

At the end of the experiment, fecal samples from 6 randomly selected rats per group were collected for detection. 16S rDNA sequencing and bioinformatics analysis were performed by Beijing Novogene Technology Co., Ltd. After extracting DNA from the fecal samples, the V3–V4 region of rDNA was amplified using barcode-specific primers (341F: CCTACGGNGNGCWGCAG; 806R: GGACTACHVGGGTATATCTAAT). Subsequently, the PCR amplicons were collected for bead purification and pooled in equimolar amounts based on their concentrations. After mixing, the PCR products were detected, and the target bands were recovered. A sequencing library was constructed, quantified by Qubit and qPCR, and subjected to sequencing on the Illumina PE250 platform after passing quality control. Following sequencing, raw data were demultiplexed to obtain sample-specific datasets based on the Barcode and PCR primer sequences. After truncating the Barcode and primer sequences, the reads of each sample were assembled using FLASH software (version 1.2.11, http://ccb.jhu.edu/software/FLASH/, accessed on 19 May 2026) to generate raw Tags data. Raw Tags were then filtered to obtain high-quality Tags, and chimeric sequences were removed to yield final valid data. Based on the valid data, operational taxonomic unit (OTU) abundance statistics, taxonomic annotation, α-diversity analysis, and β-diversity analysis were performed.

### 4.10. Reverse Transcription Quantitative Polymerase Chain Reaction (RT-qPCR)

According to the manufacturer’s instructions, total RNA was extracted from frozen hippocampal tissues using the Fast Pure Cell/Tissue Total RNA Isolation Kit V2 (Cat. No. RC112) (Vazyme Biotech Co., Ltd., Nanjing, China). cDNA synthesis was performed with the HiScript III RT SuperMix for qPCR (gDNA Wiper) (Cat. No. R323) (Vazyme Biotech Co., Ltd., Nanjing, China). Real-time quantitative PCR analysis was conducted using the ChamQ Universal SYBR qPCR Master Mix (Vazyme Biotech Co., Ltd., Nanjing, China) and the following primer sequences (Table 2), with amplification cycles carried out on a 7500 Fast Real-Time PCR System (Thermo Fisher Scientific, Waltham, MA, USA).

The PCR conditions were as follows: pre-denaturation at 95 °C for 10 min, followed by 40 cycles of denaturation at 95 °C for 15 s, annealing at 60 °C for 30 s, and extension at 72 °C for 30 s. Ribosomal protein S18 (RPS18) was used as the endogenous reference gene for each sample, and the relative gene expression levels were calculated using the 2^−∆∆Ct^ method.

### 4.11. Statistical Analysis

GraphPad Prism (version 8.0, GraphPad Software, San Diego, CA, USA) was used for statistical analysis and graphing. Experimental data were presented as mean ± standard deviation (SD). Independent samples *t*-test with Bonferroni correction was adopted for comparison among three groups, and a corrected *p* value < 0.01 was considered statistically significant. One-way analysis of variance (ANOVA) was used for comparisons of multiple groups. A *p* value < 0.05 was regarded as statistically significant.

## 5. Conclusions

The present study demonstrates that ABOL is efficacious in reducing anxiety-like behaviours induced by CRS in rats. In the context of addressing pathological injuries, ABOL has been demonstrated to reduce inflammatory factor levels in the body, thereby mitigating oxidative stress responses. Furthermore, ABOL has been shown to regulate neurotransmitter balance, alleviate neuronal damage in the hippocampus, and maintain intestinal mucosal integrity and permeability. In terms of metabolic and gut microbiota regulation, ABOL modulates CRS-induced lipid metabolism disorders by targeting key pathways such as sphingolipid metabolism (e.g., sphingomyelin, SM) and glycerophospholipid metabolism (e.g., phosphatidylcholine, PC). A study of the effects of ABOL on the structure of the gut microbiota reveals a reduction in the abundance of harmful microbes, such as *Erysipelotrichales* and *Turicibacter*, alongside an increase in beneficial taxa, including *Akkermansia* and *Muribaculaceae*. These findings provide new insights into therapeutic strategies for anxiety disorders, offering experimental data and theoretical support for the clinical application of ABOL.

## Figures and Tables

**Table 1:** Metabolites with Differential Expression.

NO.	Description	HMDB	*m*/*z*	RT	Adducts	Intensity in CON	Intensity in MOD	Intensity in ASH
1	Creatinine ^1^	HMDB0000562	114.0898	1.13	M+H	160,873.88 ± 10,531.85	173,671.63 ± 10,101.37	183,826.75 ± 7121.00
2	L-Cysteine ^1^	HMDB00574	139.0748	39.92	M+NH_4_	345,515.38 ± 12,691.62	366,475.88 ± 11,968.24	388,720.38 ± 17,904.23
3	Cysteinylglycine ^1^	HMDB0000078	179.0918	35.64	M+H	102,019.63 ± 2060.46	109,306.75 ± 3642.16	120,520.00 ± 3467.59
4	3-Methyladipoylcarnitine ^1^	HMDB0240666	245.0786	11.65	M+H	112,435.38 ± 5595.56	122,395.88 ± 6093.27	139,029.50 ± 10,120.17
5	(±)-11-Methylhexadecanoic acid ^1^	HMDB0040598	271.275	34.44	M+H	114,089.50 ± 5283.28	121,668.00 ± 5883.85	140,579.88 ± 8919.44
6	Dodeca-6,8,10-trienoylcarnitine ^1^	HMDB0241278	279.1597	31.22	M+H	222,859.38 ± 9049.22	237,702.88 ± 8986.96	253,547.38 ± 8071.40
7	Alpha-Linolenoyl ethanolamide ^1^	HMDB0013624	304.2998	19.4	M+H-H_2_O	256,384.88 ± 14,320.79	271,028.75 ± 11,123.65	289,515.75 ± 13,828.29
8	Arachidonic acid ^1^	HMDB0001043	305.2491	32.84	M+H	300,377.63 ± 23,085.84	325,134.13 ± 21,471.45	359,444.75 ± 25,498.20
9	6-Methylheptadecanoylcarnitine ^1^	HMDB0240879	369.2999	34.13	M+H	52,748.50 ± 3223.39	59,538.13 ± 4774.90	74,198.63 ± 5169.72
10	LysoPA (i-14:0/0:0) ^1^	HMDB0114765	383.2011	25.31	M+H	90,391.25 ± 3994.83	96,556.63 ± 3391.36	112,781.88 ± 6383.82
11	O-(17-Carboxyheptadecanoyl)carnitine ^1^	HMDB0240777	399.2502	31.22	M+H	223,912.38 ± 4862.81	236,411.38 ± 11,952.26	251,337.38 ± 6789.32
12	(5Z,8Z,11Z,13E,15S)-15-Hydroperoxyicosa-5,8,11,13-tetraenoylcarnitine ^1^	HMDB0241880	421.2338	33.15	M+H	50,645.25 ± 4184.62	60,831.38 ± 5513.46	78,631.50 ± 7290.46
13	LysoPA (18:2(9Z,12Z)/0:0) ^1^	HMDB0007856	435.2139	20.39	M+H	711,494.00 ± 43,712.91	754,054.25 ± 28,829.16	839,288.88 ± 49,193.26
14	Taurolithocholic acid 3-sulfate ^1^	HMDB0002580	564.2223	36.03	M+H	730,235.63 ± 32,590.27	798,363.13 ± 34,777.55	848,632.75 ± 46,167.29
15	LysoPC (24:0/0:0) ^1^	HMDB0010405	608.4664	33.71	M+H	12,955.88 ± 6658.63	22,226.75 ± 10,114.73	38,980.50 ± 11,482.80
16	Oxidized glutathione ^2^	HMDB0003337	611.1425	2.72	M-H	15,268.25 ± 6421.48	8434.88 ± 3323.47	4363.63 ± 1356.46
17	SM (d18:1/24:0) ^1^	HMDB0011697	815.6969	33.77	M+H	43,315.75 ± 11,437.29	25,837.13 ± 6487.91	37,701.63 ± 4857.06
18	PC (24:0/14:0) ^1^	HMDB0008755	835.6727	34.89	M+NH4	13,460.88 ± 4560.68	6482.88 ± 1853.40	15,169.00 ± 2701.76
19	Imidazolelactic acid ^2^	HMDB0002320	215.0342	0.98	M+HAC-H	205,473.88 ± 55,057.90	119,738.13 ± 35,803.56	170,905.75 ± 22,369.83
20	DG (16:0/0:0/PGD1) ^2^	HMDB0295589	221.1553	21.44	M-3H	25,837.25 ± 1563.69	33,545.25 ± 2980.58	49,097.50 ± 4123.11
21	Palmitic acid ^2^	HMDB0000220	255.2333	34.82	M-H	534,833.63 ± 80,806.95	501,065.38 ± 93,709.03	443,732.13 ± 95,108.03
22	DL-Homocystine ^2^	HMDB0000575	289.0696	11.58	M+2NA-H	232,176.50 ± 5181.80	255,358.75 ± 5433.63	283,532.63 ± 11,752.58
23	TG (15:0/20:0/20:2n6) ^2^	HMDB0043092	299.2589	28.55	M-3H	104,753.50 ± 10,730.79	143,740.63 ± 8664.80	133,269.88 ± 9378.62
24	LacCer (d18:1/20:0) ^2^	HMDB0011593	304.9148	21.7	M-3H	51,627.38 ± 3570.45	58,916.50 ± 3447.29	66,345.75 ± 3301.47
25	4-Hydroxybutyric acid ^2^	HMDB0000710	311.1265	7.99	3M-H	51,123.00 ± 5338.94	54,753.25 ± 4229.91	52,763.13 ± 4169.22
26	Docosapentaenoic acid (22n-3) ^2^	HMDB0006528	329.2329	11.95	M-H	89,114.88 ± 4841.43	96,085.38 ± 3290.98	109,849.25 ± 7497.89
27	DG (18:3n3/0:0/22:5n3) ^2^	HMDB0056370	331.2486	13.91	M-2H	48,138.13 ± 3014.17	50,035.75 ± 3256.77	53,404.00 ± 2274.93
28	PA (18:1(12Z)-2OH(9,10)/14:1(9Z) ^2^	HMDB0263113	337.2088	33.06	M-2H	14,832.88 ± 7224.14	5831.50 ± 2779.34	2671.25 ± 228.98
29	PA (22:5(4Z,7Z,10Z,13Z,19Z)-O(16,17)/i-12:0) ^2^	HMDB0267402	339.2016	31.83	M-2H	41,109.00 ± 11,820.46	28,231.75 ± 4648.61	18,760.88 ± 1164.78
30	8-Isoprostaglandin F2a ^2^	HMDB0005083	353.2023	25.7	M-H	75,582.50 ± 5150.67	90,994.25 ± 7396.26	77,405.25 ± 6987.50
31	Thyrotropin releasing hormone ^2^	HMDB0060080	361.1641	14.21	M-H	187,477.38 ± 43,009.74	142,390.25 ± 34,167.79	180,860.13 ± 36,589.66
32	all-trans-Retinoic acid ^2^	HMDB0001852	367.1886	27.93	M-H+HCOONa	199,346.38 ± 11,530.07	254,621.88 ± 16,254.93	291,908.63 ± 15,073.79
33	Leukotriene B4 ^2^	HMDB0001085	371.2251	33.39	M+cl	93,197.38 ± 11,992.92	77,554.00 ± 15,684.00	102,381.75 ± 21,469.75
34	MG (18:0/0:0/0:0) ^2^	HMDB0011131	395.2195	33.04	M+K-2H	35,378.00 ± 7495.97	23,779.75 ± 5455.68	34,014.63 ± 10,721.43
35	LysoPC (16:0/0:0) ^2^	HMDB0010382	608.3186	24.04	M+TFA-H	652,633.38 ± 66,993.36	793,218.75 ± 104,591.33	941,363.75 ± 138,884.28
36	PC (20:3(6,8,11)-OH(5)/2:0) ^2^	HMDB0288901	624.2903	24.03	M+Na-2H	17,087.50 ± 5019.96	28,971.38 ± 4962.51	35,404.25 ± 4165.96
37	LysoPC (0:0/18:0) ^2^	HMDB0011128	636.348	30.94	M+TFA-H	526,286.13 ± 30,481.65	602,984.13 ± 47,521.53	683,094.00 ± 79,418.20
38	PE-NMe (14:1(9Z)/14:1(9Z)) ^2^	HMDB0112942	724.3065	21.62	M+Br	30,769.63 ± 4599.45	43,112.00 ± 7472.53	53,028.88 ± 8718.02
39	11-trans-Leukotriene C4 ^2^	HMDB0005095	738.277	23.91	M+TFA-H	38,605.25 ± 6046.01	60,045.88 ± 8100.34	77,079.38 ± 4840.68

Note: ^1^: Positive ion mode; ^2^: Negative ion mode.

**Table 2:** Primer Sequences.

Gene	Forward Primer (5′-3′)	Reverse Primer (5′-3′)
SGMS1	ATAGAGGTGGCCTTGTTTCTGG	GGTGTGAACTATGTGGGCTGTA
SGMS2	CAGAAGATCGGGGAGGATAACG	TTCACTGCTCCAATCTTTTGCG
CERS2	GAAACAGAGAGTTCAGAGGGGG	TATCCTTTCTCCCCAGAGCTCA
SMPD1	ATCAGGTGTCTCCAGAGCTACT	GGTAAGGACCTCTCCAGTCTCT
CEPT	AGAACCAAGACGCCTAAGGAAG	CTGTACAAGCTAGCCAGGTGAA
LPCAT3	CTGAAGACTATGATACCCGCCC	ACCCAGGCATTGGTATTGATGT

## Data Availability

The original contributions presented in this study are included in the article. Further inquiries can be directed to the corresponding authors.

## Supplementary Materials

The following supporting information can be downloaded at: https://www.mdpi.com/article/10.3390/ph19060831/s1, Supplementary Material File S1: (1). Preparation of *Anshen Bunao* Oral Liquid (ABOL); (2). Pharmacodynamic Material Basis of ABOL [57]; (3). Supplementary histopathological figures; Figure S1: HE staining of the CA3 region of the brain; Figure S2: Nissl staining of the DG region of the brain; Figure S3: Immunohistochemical staining of ZO 1 protein; Figure S4: Immunohistochemical staining of Caludin-1 protein; Supplementary Material File S2: HPLC fingerprint of ABOL [59].

## Abbreviations

5-HT	5-hydroxytryptamine
ABOL	Anshen Bunao Oral Liquid
ASM	Acid sphingomyelinase
CEPT	Choline/ethanolamine phosphotransferase
CERS2	Ceramide synthase 2
CNS	Central nervous system
CORT	Corticosterone
CRS	Chronic restraint stress
EPM	Elevated-plus maze test
GABA	γ-aminobutyric acid
HPA	Hypothalamic–pituitary–adrenal
LPCAT3	Lysophosphatidylcholine acyltransferase 3
NE	Norepinephrine
OFT	Open Field Test
OPLS-DA	Orthogonal partial least squares-discriminant analysis
PC	Phosphatidylcholine
PCA	Principal component analysis
PLA2G6	Phospholipase A2 group VI
QC	Quality control
SCFAs	Short-chain fatty acids
SGMS	Sphingomyelin synthase
SM	Sphingomyelin
SPT	Sucrose Preference Test
ZO-1	Tight junction protein 1
