# Supplementary material for: Gut Microbiota and Metabolite Remodeling Underlies the Anxiolytic Effect of Anshen Bunao Oral Liquid
Source: Pharmaceuticals (Basel). 2026 May 26;19(6):831. doi: 10.3390/ph19060831 (PMC13305180; doi:10.3390/ph19060831)
Supplement: Supplementary file 1 [file pharmaceuticals-19-00831-s001.zip › Supplementary Material File S1.pdf]

Supplementary Material

(1).Preparation of Anshen Bunao Oral Liquid (ABOL)

S1.1 Composition and Content Proportions

Cornu Cervi Pantotrichum 3g, Polygoni Multiflori Preparata 62.5g, Epimedii Folium 50g, Rhizoma Zingiberis 12.5g, Radix Glycyrrhizae 6.25g, Fructus Jujubae 12.5g, Vitamin B_1 0.5g.

S1.2 Preparation Process

Of the above seven ingredients, the volatile oil in Rhizoma Zingiberis is extracted by steam distillation. The residue is decocted with water together with Radix Polygoni Multiflori Preparata, Herba Epimedii, Fructus Jujubae and Radix Glycyrrhizae for three times. The decoctions are combined, filtered and concentrated to an appropriate volume. Three volumes of ethanol are added, and the mixture is allowed to stand, filtered, and the filtrate is reserved for further use.

Cornu Cervi Pantotrichum is decocted with water for five times. The decoctions are filtered and combined, then concentrated. Beeswax is added, and the solution is allowed to stand until the wax layer is completely solidified; the wax layer is then removed, followed by suction filtration. Ethanol is added to adjust the alcoholicity to 80%, the mixture is allowed to stand and filtered, and ethanol is recovered from the filtrate, which is then concentrated to an appropriate volume. Ethanol is added again to adjust the alcoholicity to 75%, the mixture is allowed to stand and filtered, and ethanol is recovered from the filtrate. Water and ethanol are added to adjust the alcoholicity to 20%–30%, yielding the Cornu Cervi Pantotrichum extract.

The above medicinal liquid, the Cornu Cervi Pantotrichum extract and the aqueous sucrose solution (containing 180 g of sucrose) are combined and mixed homogeneously. The volatile oil of Rhizoma Zingiberis, Vitamin B_1, benzoic acid, sodium benzoate and ethylparaben are added successively. The mixture is stirred uniformly, allowed to stand, filtered, and water is added to make the total volume up to 1000 mL. Homogenization gives the final preparation.

(2). Pharmacodynamic Material Basis of Anshen Bunao Oral Liquid

S2.1 Preparation of Anshen Bunao Oral Liquid Test Solution

One milliliter of ABOL was filtered through a 0.22 μm microporous membrane, and the subsequent filtrate was collected for subsequent use.

S2.2 UPLC-Q/TOF MS) was employed for the systematic identification of chemical constituents in Anshen Bunao Oral Liquid (ABOL).

S2.2.1 Method

Chromatographic Conditions:

A Waters Acquity UPLC system coupled with an Acquity UPLC HSS T3 column (2.1×100 mm, 2.5 μm) was utilized for chromatographic separation. The mobile phase consisted of 0.1% formic acid in water (Phase A) and methanol (Phase B) with gradient elution. The column temperature was set at 30 °C, and the injection volume was 2 μL. The gradient elution program was as follows: 100% A at 0–3 min; linear gradient from 100% A to 72% A at 3–11 min; linear gradient from 72% A to 67% A at 11–13 min; linear gradient from 67% A to 50% A at 13–18 min; linear gradient from 50% A to 46% A at 18–21 min; linear gradient from 46% A to 27% A at 21–32 min; linear gradient from 27% A to 2% A at 32–35 min; and 2% A maintained at 35–37 min.

Mass Spectrometric Conditions:

An AB Sciex Q/TOF MS 5600 mass spectrometer was adopted, with the parameter settings as follows: the acquisition time was 37 min; the mass range was set at 50–2000 Da; the ion source temperature was 500 °C; the ion spray voltage floating (ISVF) was 5500 V in positive ion mode and 4500 V in negative ion mode; the curtain gas (CUR) was 35 psi, the nebulizer gas (GS1) and auxiliary gas (GS2) were both set at 50 psi; the declustering potential (DP) was 80 V, and the collision energy was configured as 10 V for MS^1 and 40±20 V for MS^2. For real-time mass calibration, the internal standard ion at m/z 609.28 was selected in positive ion mode, while the internal standard ion at m/z 403.11 was adopted in negative ion mode. The raw data were collected in information-dependent acquisition (IDA) mode via the Analyst® TF Workstation (Version 1.6, AB SCIEX, USA).

S2.2.2 qualitative compound identification

Establishment of Chemical Constituent Library: Chemical constituent information (including molecular formula, molecular structural formula, relative molecular weight, etc.) of Cornu Cervi Pantotrichum, Polygonum multiflorum Preparata, Epimedii Folium, Radix Glycyrrhizae, Rhizoma Zingiberis and Fructus Jujubae was obtained via the Traditional Chinese Medicine Systems Pharmacology Database and Analysis Platform (TCMSP, https://old.tcmsp-e.com/tcmsp.php), the HERB database (https://www.drugsnav.com/detail/tuijian/528.html) and literature retrieval. The

chemical constituents of each medicinal material were collated with duplicate removal.
Mass Spectrometric Data Processing: Raw mass spectrometric data were imported into PeakView software (Version 1.2, AB SCIEX, USA) for data processing including retention time calibration, peak identification, data extraction, alignment and integration. The accurate quasi-molecular ion peaks in MS^1 were used to analyze the elemental composition and potential molecular formulas of unknown compounds via the "Formula Finder" plug-in, and chemical formulas with a mass error within 10 ppm were screened out. Subsequently, the matching chemical constituents were retrieved from the chemical constituent database of Anshen Bunao Liquid, and their mol files were compared and analyzed with the fragment ion information in MS^2 for the accurate identification of the chemical constituents.

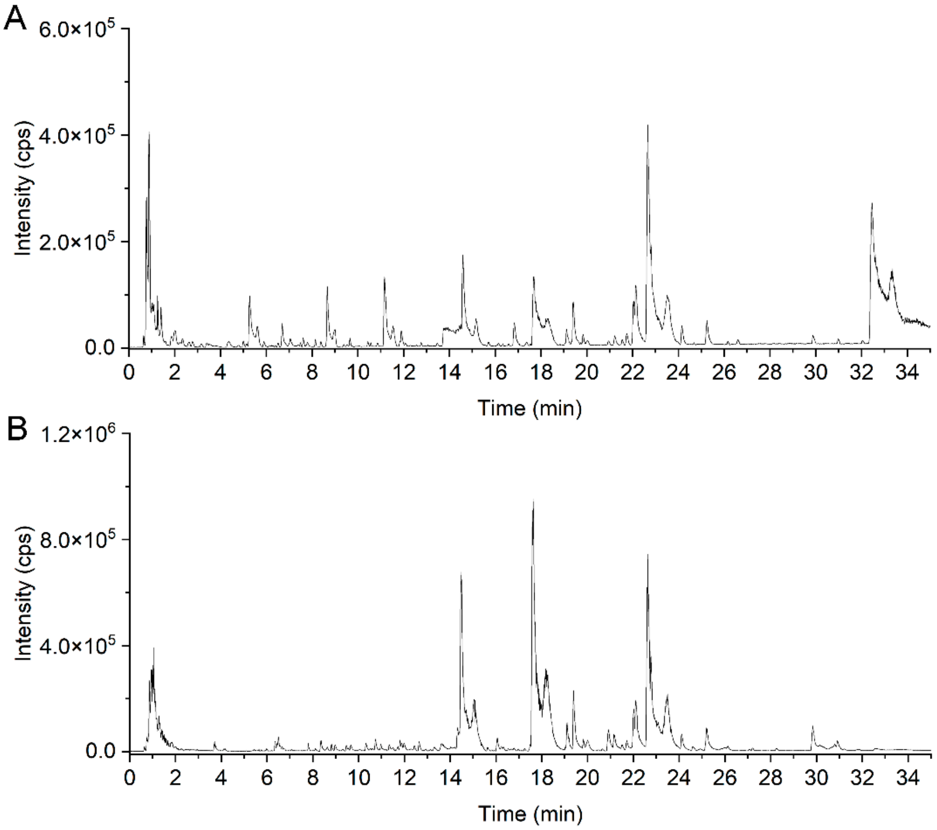

The base peak chromatogram of Anshen Bunao syrup on positive ion mode (A) and negative ion mode (B). Note: cps, counts per second

(3). Supplementary histopathological figures

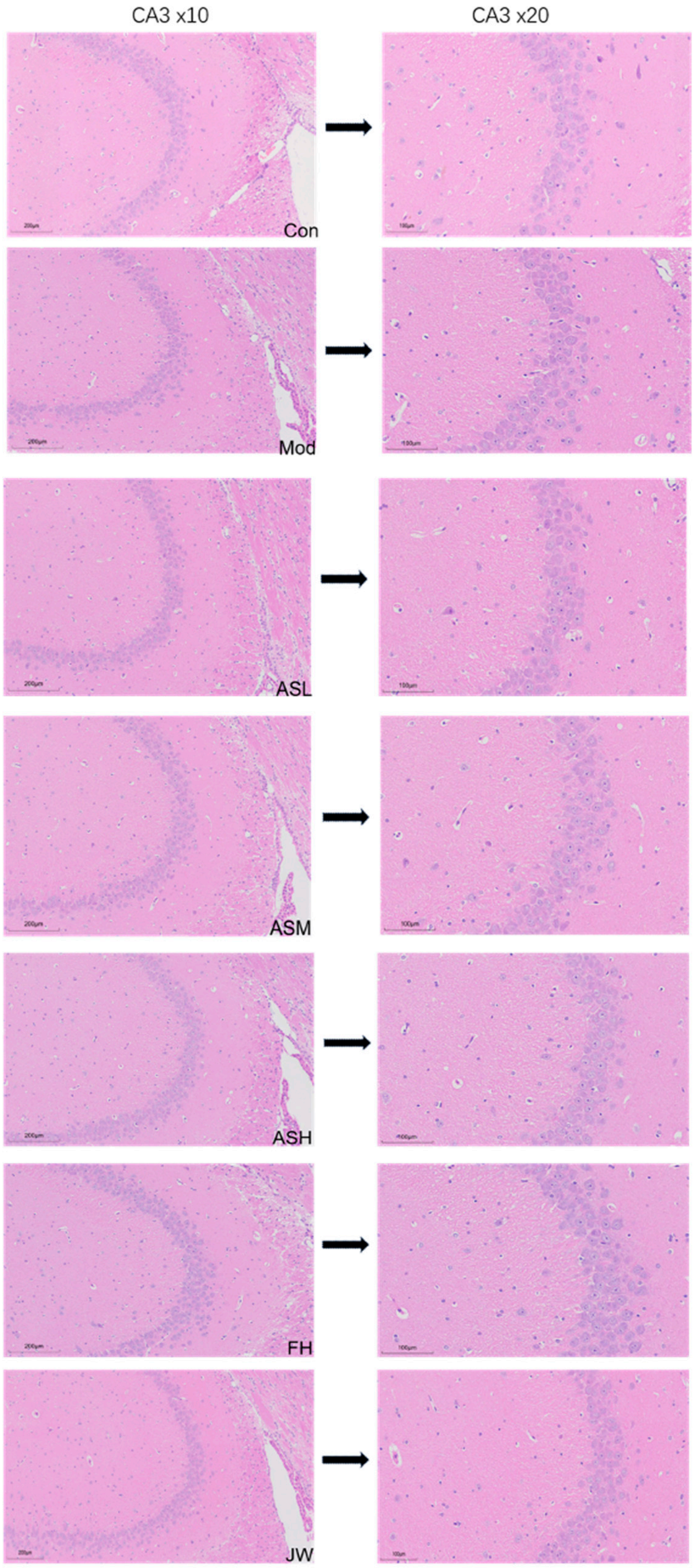

Figure S1 HE staining of the CA3 region of the brain

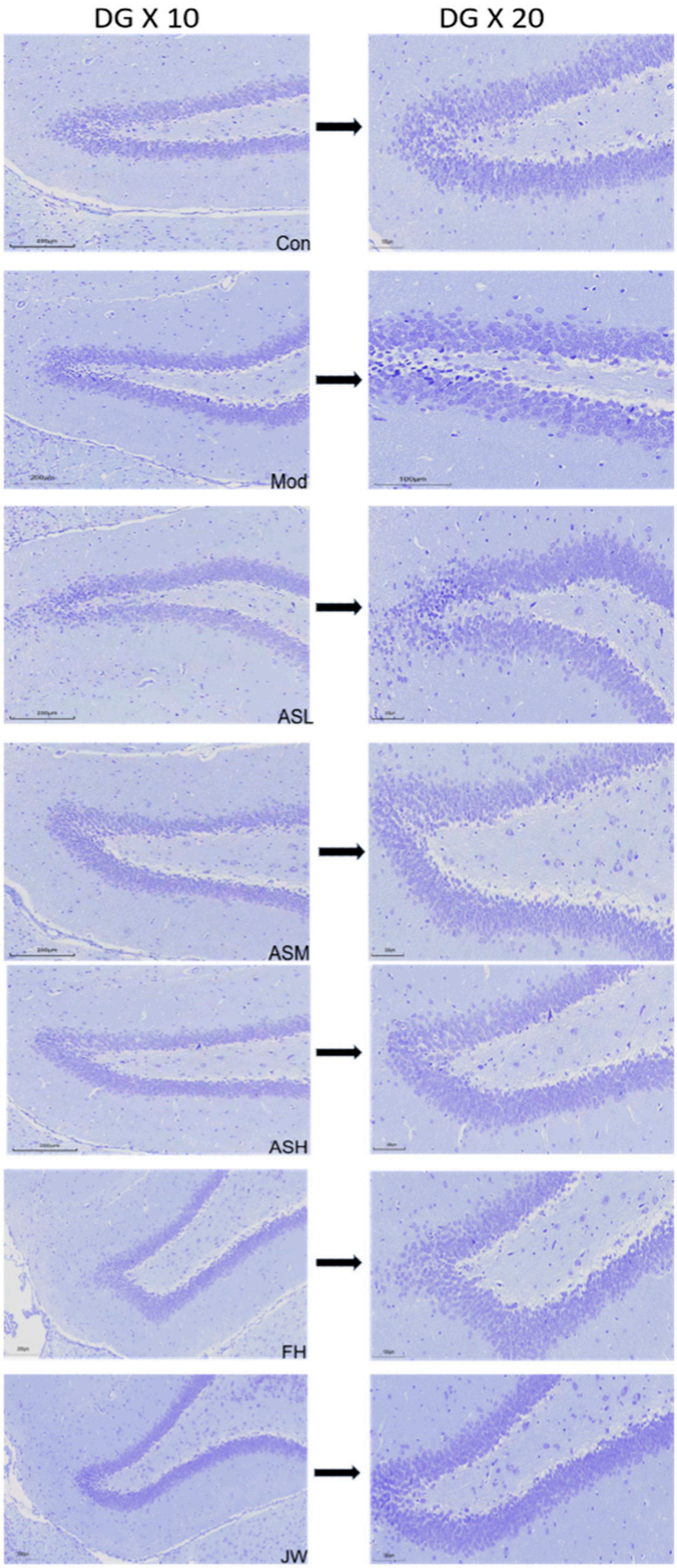

Figure S2 Nissl staining of the DG region of the brain

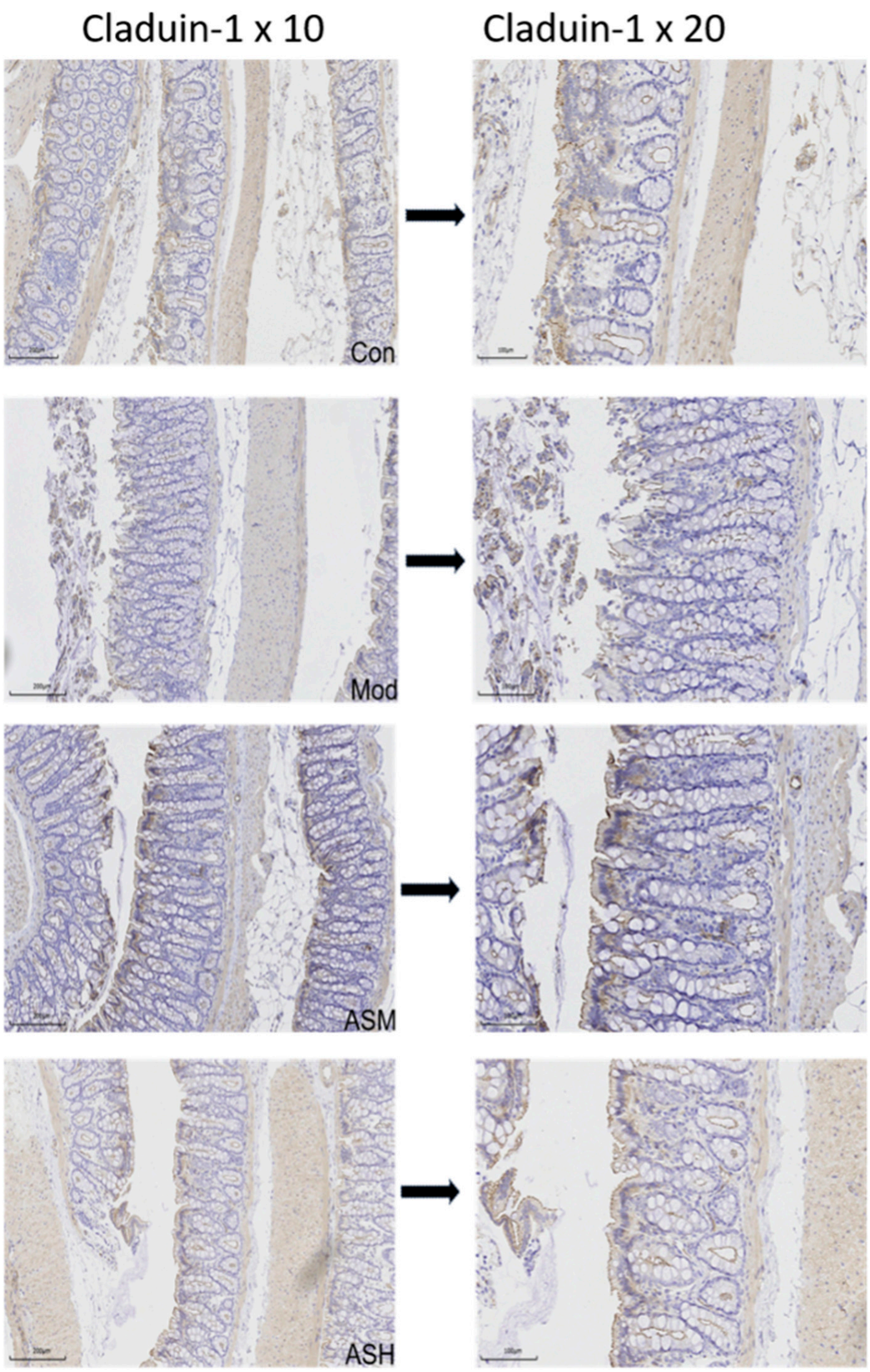

Figure S3 Immunohistochemical staining of ZO-1 protein

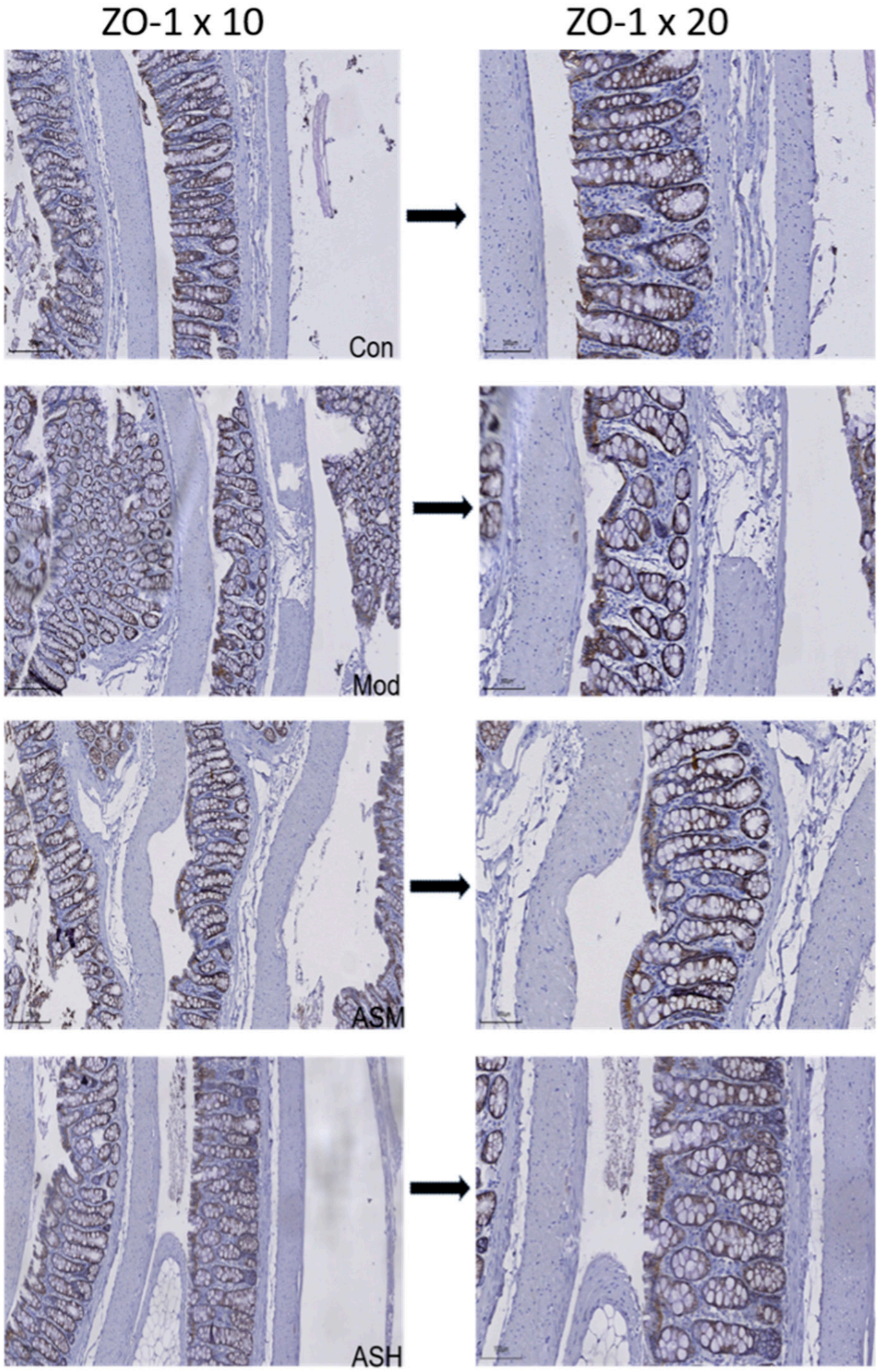

Figure S4 Immunohistochemical staining of Caludin-1 protein
