# Supplementary material for: Gut Microbiota and Metabolite Remodeling Underlies the Anxiolytic Effect of Anshen Bunao Oral Liquid
Source: Pharmaceuticals (Basel). 2026 May 26;19(6):831. doi: 10.3390/ph19060831 (PMC13305180; doi:10.3390/ph19060831)
Supplement: Supplementary file 1 [file pharmaceuticals-19-00831-s001.zip › Supplementary Material File S2.pdf]

HPLC fingerprint of ABOL

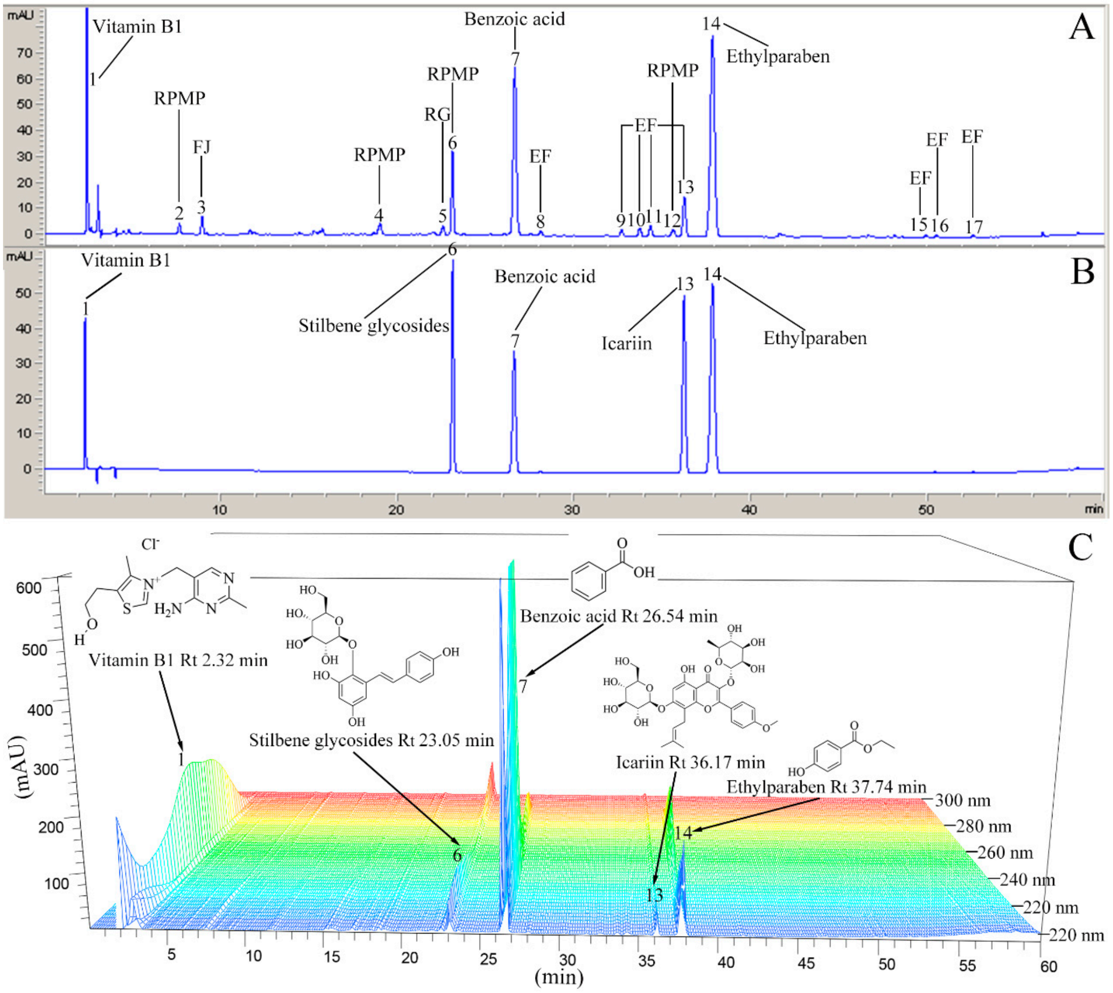

(A) Representative HPLC chromatogram of ABOL recorded at 270 nm, (B) HPLC chromatogram of the mixture standard solutions; (C) Three-dimensional HPLC fingerprint profile of ABOL.

VA, Velvet antler; PPM, Processed Polygonum multiflorum; GI, Ginger; GL, Glycyrrhiza; JU, Jujube; EP, Epimedium.

Huang *et al.* 2026_Phytomedicine (in press)
